# Supplementary material for: Slow vibrational relaxation drives ultrafast formation of photoexcited polaron pair states in glycolated conjugated polymers
Source: Nat Commun. 2024 Jul 22;15:6153. doi: 10.1038/s41467-024-50530-7 (PMC11263616; doi:10.1038/s41467-024-50530-7)
Supplement: Supplementary file 1 — Supplementary Information [file 41467_2024_50530_MOESM1_ESM.pdf]

Supplementary information

Slow vibrational relaxation drives ultrafast formation of photoexcited polaron pair states in glycolated conjugated polymers

Katia Pagano,[1,9] Jin Gwan Kim,[2,9] Joel Luke,[1] Ellasia Tan,[1] Katherine Stewart,[1] Igor V. Sazanovich,[3] Gabriel Karras,[3] Hristo I. Gonev,[4] Adam V. Marsh,[5] Na Yeong Kim,[2] Sooncheol Kwon,[6] Young Yong Kim,[7] Maria I. Alonso,[8] Bernhard Dörling,[8] Mariano Campoy-Quiles,[8] Anthony W. Parker,[3] Tracey M. Clarke,[4] Yun-Hi Kim,[2] and Ji-Seon Kim[1*]*

[1] Department of Physics and Centre for Processable Electronics, Imperial College London, London, SW7 2AZ, United Kingdom

[2] Department of Chemistry and Research Institute of Molecular Alchemy (RIMA) Gyeongsang National University Jinju, Gyeongnam 660–701, South Korea

[3] Central Laser Facility, Research Complex at Harwell, STFC Rutherford Appleton Laboratory, Didcot, Oxfordshire OX11 0QX, United Kingdom

[4] Department of Chemistry, University College London, Christopher Ingold Building, London WC1H 0AJ, United Kingdom

[5] Physical Science and Engineering Division, KAUST Solar Center (KSC), King Abdullah University of Science and Technology (KAUST), Thuwal 23955-6900, Saudi Arabia.

[6] Department of Energy and Materials Engineering, Dongguk University-Seoul, Seoul, 04620, Republic of Korea

[7] Beamline Division, Pohang Accelerator Laboratory, Pohang University of Science and Technology, Pohang 37673, Republic of Korea

[8] Department of Nanostructured Materials, Institut de Ciència de Materials de Barcelona, ICMAB-CSIC, E-08193 Bellaterra, Spain

[9] These authors contributed equally to the work.

Email: ji-seon.kim@imperial.ac.uk, ykim@gnu.ac.uk

Supplementary Methods: Material Synthesis

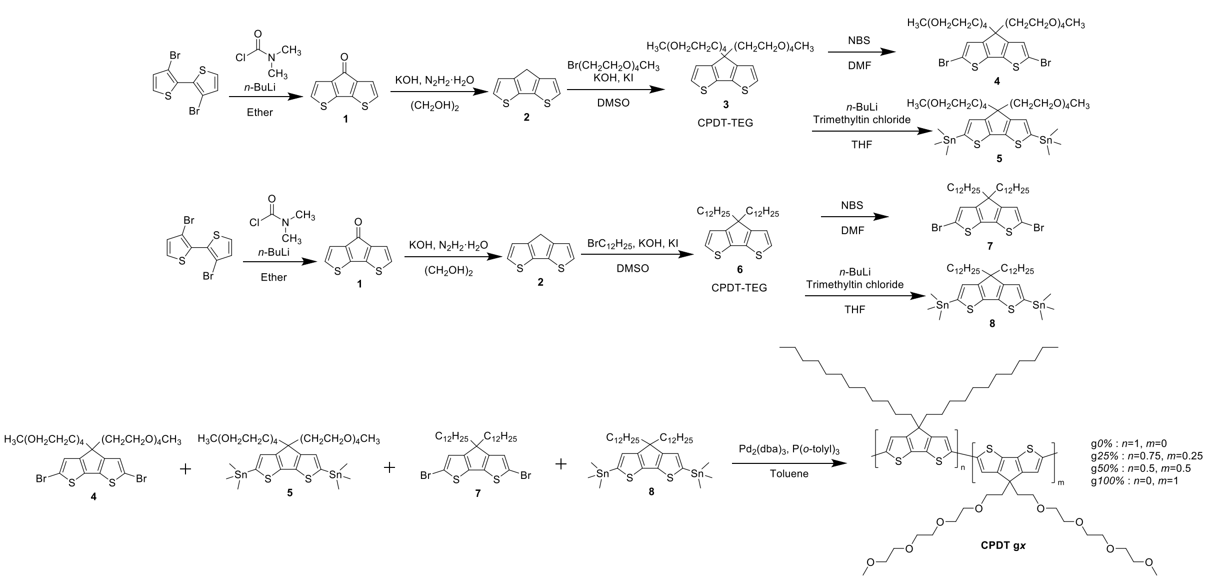

Suppl. Fig. 1: CPDT synthesis. Schematic showing the synthesis steps of CPDT g*x*.

Synthesis of 4H-cyclopenta[2,1-b:3,4-b']dithiophen-4-one (1)

3,3'-Dibromo-2,2'-bithiophene (30 g, 92.58 mmol) was dissolved in anhydrous diethyl ether (500 mL) under the nitrogen atmosphere. 88.9 of *n*-BuLi (2.5 M in *n*-hexane, 222.19 mmol) was added dropwise at -78 ℃. The reaction mixture was maintained at -78 ℃ for 2 h and then dimethylcarbamoyl chloride (10.95 g, 101.84 mmol) was added. The mixture was slowly warmed up to room temperature overnight. The reaction mixture was poured into water and extracted with diethyl ether (3 × 100 mL). The organic solution was dried over anhydrous magnesium sulfate and concentrated under reduced pressure. The crude compound was purified by column chromatography on silica gel using dichloromethane/*n*-hexane (1:1, v/v) as eluent. (Yield: 10.8 g, 61%). ^{1}H NMR (300 MHz, $CDCl_3$): δ (ppm) = 7.07 (d, 2 H, J = 4.87 Hz), 7.02 (d, 2 H, J = 4.87 Hz), ^{13}C NMR (500 MHz, $CDCl_3$): δ (ppm) = 182.67, 149.21, 142.43, 127.23, 121.17, HRMS (EI^{+}) *m/z* calcd. for $C_9H_4OS_2$ 191.9704, found 191.9709.

Synthesis of 4H-cyclopenta[2,1-b:3,4-b']dithiophene (2)

4H-cyclopenta-[2,1-b:3,4-b']dithiophen-4-one (10.0 g, 52.02 mmol) was suspended in 150 mL of ethylene glycol. Potassium hydroxide (11.67 g, 208.06 mmol) and hydrazine hydrate (76.7 ml) were added under N_2. The mixture was stirred for 24 h at 180 ℃. The resulting

solution was slowly cooled down to room temperature and washed with water. The organic layer was extracted with methylene dichloride and dried over anhydrous magnesium sulphate. After the solvent was evaporated, the crude product was purified by column chromatography using *n*-hexane as an eluent. After purification, the product was obtained as a white solid (Yield: 7.0 g, 75%). ^{1}H NMR (300 MHz, $CDCl_3$): δ (ppm) = 7.21 (d, 2 H, *J*=4.87 Hz), 7.12 (d, 2 H, *J*=4.87 Hz), 3.56 (s, 2 H), ^{13}C NMR (500 MHz, $CDCl_3$): δ (ppm) =149.72, 138.70, 124.51, 123.00, 31.86. HRMS (EI^{+}) *m/z* calcd. for $C_9H_6S_2$, 177.9911, found 177.9908.

Synthesis of 13,13'-(4H-cyclopenta[2,1-b:3,4-b']dithiophene-4,4-diyl)bis(2,5,8,11-tetraoxatridecane) (3)

To a suspension solution of 4H-cyclopenta[2,1-b:3,4-']dithiophene (3 g, 16.83 mmol) in DMSO (100 mL), potassium hydroxide (3.78 g, 67.31 mmol) and potassium iodide (0.08 g, 0.50 mmol) were added, followed by 13-bromo-2,5,8,11-tetraoxatridecane (10.04 g, 37.02 mmol). The mixture was stirred for 24 h at room temperature and under N_2 atmosphere. The reaction mixture was washed with water. The organic phase was extracted with diethyl ether, dried over magnesium sulphate and the solvent was removed under reduced pressure. The crude product was purified by column chromatography using diethyl ether/ acetone (8:1, v/v) as eluent. After purification, the product was obtained as a light-yellow oil (Yield: 6 g, 63%). ^{1}H-NMR (300 MHz, CD_2Cl_2) δ = 7.24 (d, *J* = 4.90 Hz, 1 H), 7.07 (d, *J* = 4.90 Hz, 1 H), 3.61-3.44 (m, 10 H), 3.37-3.31 (m, 5 H), 3.08 (m, 2 H), 2.30 (m, 2 H). ^{13}C NMR (500 MHz, $CDCl_3$): δ (ppm) =156.45, 136.56, 125.02, 121.71, 71.93, 70.57, 70.51, 70.08, 67.60, 59.04, 49.20, 37.56. HRMS (EI^{+}) *m/z* calcd. for $C_{27}H_{42}O_8S_2$, 558.2321; Found: 558.2329.

Synthesis of 13,13'-(2,6-dibromo-4H-cyclopenta[2,1-b:3,4-b']dithiophene-4,4-diyl)bis(2,5,8,11-tetraoxatridecane) (4)

Under a nitrogen atmosphere, 13,13'-(4H-cyclopenta[2,1-b:3,4-b']dithiophene-4,4-diyl)bis(2,5,8,11-tetraoxatridecane) (1.5 g, 2.68 mmol) was dissolved in DMF (40 mL). N-Bromosuccinimide (0.96 g, 5.37 mmol) was added to the mixture. Then the reaction mixture was stirred at room temperature for 12 h. The reaction mixture was washed with water. The organic phase was extracted with diethyl ether and dried over magnesium sulphate. After the solvent was evaporated, the crude product was purified by column chromatography using diethyl ether /acetone (8:1, v/v) as eluent. After purification, the product was obtained as a

light-yellow oil (Yield: 1.7 g, 88 %). ^{1}H-NMR (300 MHz, CD_2Cl_2) δ = 7.10 (s, 1H), 3.61-3.45 (m, 10 H), 3.37-3.32 (m, 5H), 3.10-3.05 (m, 2H), 2.25-2.20 (m, 2H). ^{13}C NMR (500 MHz, $CDCl_3$): δ (ppm) =154.64, 136.33, 124.98, 111.45, 71.94, 70.56, 70.51, 70.20, 67.37, 59.05, 51.12, 37.52. HRMS (FAB$^+$) *m/z* calcd. for $C_{27}H_{40}Br_2O_8S_2$, 714.0531; Found: 714.0519.

Synthesis of (4,4-di(2,5,8,11-tetraoxatridecan-13-yl)-4H-cyclopenta[2,1-b:3,4-b']dithiophene-2,6-diyl)bis(trimethylstannane) (5)

To a stirred solution of 13,13'-(4H-cyclopenta[2,1-b:3,4-b']dithiophene-4,4-diyl)bis(2,5,8,11-tetraoxatridecane) (1.0 g, 1.97 mmol) in anhydrous tetrahydrofuran (20 mL) at -78 ℃ under nitrogen atmosphere was added dropwise a solution of *n*-BuLi (2.5 M solution in *n*-hexane, 3.15 mL, 7.87 mmol). The reaction mixture was stirred for 2 h at -78 ℃ and then tributyltin chloride (1.0 M solution in THF, 9.84 mL, 9.84 mmol) was added. The mixture was slowly warmed up to room temperature and stirred 4 h. The reaction mixture was diluted with water and extracted with diethyl ether following dried over magnesium sulphate. After the solvent was evaporated, the crude product used directly for the next step without purification. ^{1}H NMR (300 MHz, CD_2Cl_2): δ (ppm) = 6.87 (s, 1 H), 3.40-3.25 (m, 10 H), 3.16-3.12 (m, 5 H), 2.87 (t, 2 H), 2.09 (t, 2 H), 0.31-0.12 (m, 9 H).

Synthesis of 4,4-didodecyl-4H-cyclopenta[2,1-b:3,4-b']dithiophene (6)

To a suspension solution of 4H-cyclopenta[2,1-b:3,4-']dithiophene (3 g, 16.83 mmol) in DMSO (100 mL), potassium hydroxide (3.78 g, 67.31 mmol) and potassium iodide (0.08 g, 0.50 mmol) were added, followed by 1-bromododecane (9.23 g, 37.02 mmol). The mixture was stirred for 24 h at room temperature and under N_2 atmosphere. The reaction mixture was washed with water and *n*-hexane. The organic phase was extracted, dried over magnesium sulphate and the solvent was removed under reduced pressure. The crude product was purified by column chromatography using *n*-hexane as eluent. After purification, the product was obtained as a light-yellow oil (Yield 5.8 g, 67%). ^{1}H-NMR (300 MHz, CD_2Cl_2) δ = 7.21 (d, J = 4.87 Hz, 1 H), 7.00 (d, J = 4.87 Hz, 1 H), 1.89-1.83 (m, 2 H), 1.33-1.16 (m, 18 H), 1.00-0.89 (m, 5 H) . ^{13}C NMR (500 MHz, $CDCl_3$): δ (ppm) =158.16, 136.46, 124.41, 121.66, 53.27, 37.75, 31.95, 30.05, 29.65, 29.61, 29.42, 29.37, 24.55, 22.72, 14.14. HRMS (EI$^+$) *m/z* calcd. for $C_{33}H_{54}S_2$, 514.3667; Found: 514.3664

Synthesis of 2,6-dibromo-4,4-didodecyl-4H-cyclopenta[2,1-b:3,4-b']dithiophene (7)

Under a nitrogen atmosphere, 4,4-didodecyl-4H-cyclopenta[2,1-b:3,4-b']dithiophene (1.5 g, 2.91 mmol) was dissolved in DMF(40 mL). N-Bromosuccinimide (1.04 g, 5.83 mmol) was added to the mixture. Then the reaction mixture was stirred at room temperature for 12 h. After the reaction, the mixture was extracted with *n*-hexane. The organic layer was washed with water and then dried over $MgSO_4$. After the solvent was evaporated, the crude product was purified by column chromatography using *n*-hexane as eluent. After purification, the product was obtained as a light-yellow oil (Yield: 1.5 g, 76 %). ^{1}H-NMR (300 MHz, CD_2Cl_2) δ = 7.01 (s, 1H), 1.85-1.79 (m, 2 H), 1.38-1.34 (m, 18 H), 0.94-0.89 (m, 5H). ^{13}C NMR (500 MHz, $CDCl_3$): δ (ppm) = 155.96, 136.31, 124.60, 111.09, 55.05, 37.55, 34.06, 32.87, 31.94, 29.95, 29.65, 29.61, 28.80, 24.44, 22.71, 14.14. HRMS (FAB$^+$) *m/z* calcd. for $C_{33}H_{52}Br_2S_2$, 670.1877; Found: 670.1876.

Synthesis of (4,4-didodecyl-4H-cyclopenta[2,1-b:3,4-b']dithiophene-2,6-diyl)bis(trimethylstannane) (8)

To a stirred solution of 13,13'-(4H-cyclopenta[2,1-b:3,4-b']dithiophene-4,4-diyl)bis(2,5,8,11-tetraoxatridecane) (1.0 g, 1.94 mmol) in anhydrous tetrahydrofuran (20 mL) at -78 ℃ under nitrogen atmosphere was added dropwise a solution of *n*-BuLi (2.5 M solution in *n*-hexane, 3.11 mL, 7.77 mmol). The reaction mixture was stirred for 2 h at -78 ℃ and then tributyltin chloride (1.0 M solution in THF, 9.71 mL, 9.71 mmol) was added. The mixture was slowly warmed up to room temperature and stirred 4 h. The reaction mixture was diluted with water and extracted with diethyl ether following dried over magnesium sulphate. After the solvent was evaporated, the crude product used directly for the next step without purification. ^{1}H NMR (300 MHz, CD_2Cl_2): δ (ppm) = 6.83 (s, 1 H), 1.66-1.60 (m, 2 H), 1.16-0.95 (m, 18 H), 0.86-0.69 (m, 5 H), 0.31-0.12 (m, 9 H).

General procedure of polymerization

Under a nitrogen atmosphere, dibromo-substituted monomer (0.357 mmol) and bis(trimethylstannyl)-substituted monomer (0.357 mmol) was dissolved in toluene (2 mL), and then deoxygenated with nitrogen for 20 min. $Pd_2(dba)_3$ (7 mol%) and $P(o\text{-}tol)_3$ (18 mol%) were

added to the mixture, and heated at 100 ℃ for 72 h. Afterwards, 2-(tributylstannyl)thiophene (0.1 mL) was added to the mixture for end-capping, and stirred for 3h. The reaction mixture was precipitated into methanol or *n*-hexane (300 mL with 0.2 M HCl aq). The crude polymer was filtered and purified by soxhlet extraction with methanol, acetone, *n*-hexane and toluene.

Synthesis of CPDT g0%

The general procedure described above using 2,6-dibromo-4,4-didodecyl-4H-cyclopenta[2,1-b:3,4-b']dithiophene (0.24 g, 0.357 mmol) and (4,4-didodecyl-4H-cyclopenta[2,1-b:3,4-b']dithiophene-2,6-diyl)bis(trimethylstannane) (0.3 g, 0.357 mmol) produced CPDT-TEG(0)-12. After the reaction, the reaction mixture was precipitated into methanol (300 mL with 0.2 M HCl aq). The crude polymer was filtered and purified by soxhlet extraction with methanol, acetone, *n*-hexane and toluene (Yield: 0.17 g, 46%). Mn = 22 kg mol-1, Mw = 49 kg mol-1, Đ = 2.22. ^{1}H-NMR (500 MHz, tetrachloroethane) δ = 7.18 (br, 1 H), 2.05 (br, 2 H), 1.35 (br, 20 H), 0.97 (br, 3 H).

Synthesis of CPDT g25%

The general procedure described above using 2,6-dibromo-4,4-didodecyl-4H-cyclopenta[2,1-b:3,4-b']dithiophene (0.12 g, 0.178 mmol), (4,4-didodecyl-4H-cyclopenta[2,1-b:3,4-b']dithiophene-2,6-diyl)bis(trimethylstannane) (0.30 g, 0.357 mmol) and 13,13'-(2,6-dibromo-4H-cyclopenta[2,1-b:3,4-b']dithiophene-4,4-diyl)bis(2,5,8,11-tetraoxatridecane) (0.13 g, 0.178 mmol) produced CPDT-TEG(0.25)-12. After the reaction, the reaction mixture was precipitated into methanol (300 mL with 0.2 M HCl aq). The crude polymer was filtered and purified by soxhlet extraction with methanol, acetone, *n*-hexane and toluene (Yield: 0.14 g, 37%). Mn = 22 kg mol^{-1}, Mw = 50 kg mol^{-1}, Đ = 2.30. ^{1}H-NMR (500 MHz, tetrachloroethane) δ = 7.16 (br, 1 H), 3.66-3.37 (br, 4.25 H), 2.38 (br, 0.5 H), 1.99 (br, 1.5 H), 1.35 (br, 15 H), 0.98 (br, 2.25 H).

Synthesis of CPDT g50%

The general procedure described above using (4,4-didodecyl-4H-cyclopenta[2,1-b:3,4-b']dithiophene-2,6-diyl)bis(trimethylstannane) (0.15 g, 0.176 mmol), 2,6-dibromo-4,4-didodecyl-4H-cyclopenta[2,1-b:3,4-b']dithiophene (0.12 g, 0.176 mmol), (4,4-di(2,5,8,11-tetraoxatridecan-13-yl)-4H-cyclopenta[2,1-b:3,4-b']dithiophene-2,6-

diyl)bis(trimethylstannane) (0.16 g, 0.176 mmol) and 13,13'-(2,6-dibromo-4H-cyclopenta[2,1-b:3,4-b']dithiophene-4,4-diyl)bis(2,5,8,11-tetraoxatridecane) (0.13 g, 0.176 mmol) produced CPDT-TEG(0.5)-12. After the reaction, the reaction mixture was precipitated into methanol (300 mL with 0.2 M HCl aq). The crude polymer was filtered and purified by soxhlet extraction with methanol, acetone, *n*-hexane and toluene (Yield: 0.2 g, 52%). *M*n = 23 kg mol^{-1}, *M*w = 55 kg mol^{-1}, Đ = 2.37. ^{1}H-NMR (500 MHz, tetrachloroethane) δ = 7.19 (br, 1 H), 3.66-3.37 (br, 8.5 H), 2.39 (br, 1 H), 1.99 (br, 1 H), 1.35 (br, 10 H), 0.98 (br, 1.5 H).

Synthesis of CPDT g100%

Under a nitrogen atmosphere, 13,13'-(2,6-dibromo-4H-cyclopenta[2,1-b:3,4-b']dithiophene-4,4-diyl)bis(2,5,8,11-tetraoxatridecane) (0.3 g, 0.419 mmol), 13,13'-(4H-cyclopenta[2,1-b:3,4-b']dithiophene-4,4-diyl)bis(2,5,8,11-tetraoxatridecane) (0.23 g, 0.419 mmol), $CsCO_3$ (0.44 g, 1.256 mmol) and PivOH (0.043 g, 0.419 mmol) was dissolved in toluene (4 mL), and then deoxygenated with nitrogen for 20 min. $Pd_2(dba)_3$ (0.031 g, 8 mol%) and P(*o*-OMePh)$_3$ (0.047 g, 32 mol%) were added to the mixture, and heated at 100 ℃ for 72 h. The reaction was cooled at room temperature and the polymer was precipitated in *n*-hexane (300 mL with 0.2 M HCl aq). The crude polymer was filtered and purified by soxhlet extraction with *n*-hexane and toluene (Yield: 0.2 g, 42%). *M*n = 20 kg mol^{-1}, *M*w = 66 kg mol^{-1}, Đ = 3.36. ^{1}H-NMR (500 MHz, tetrachloroethane) δ = 7.20 (br, 1 H), 3.67-3.36 (br, 17 H), 2.37 (br, 2 H).

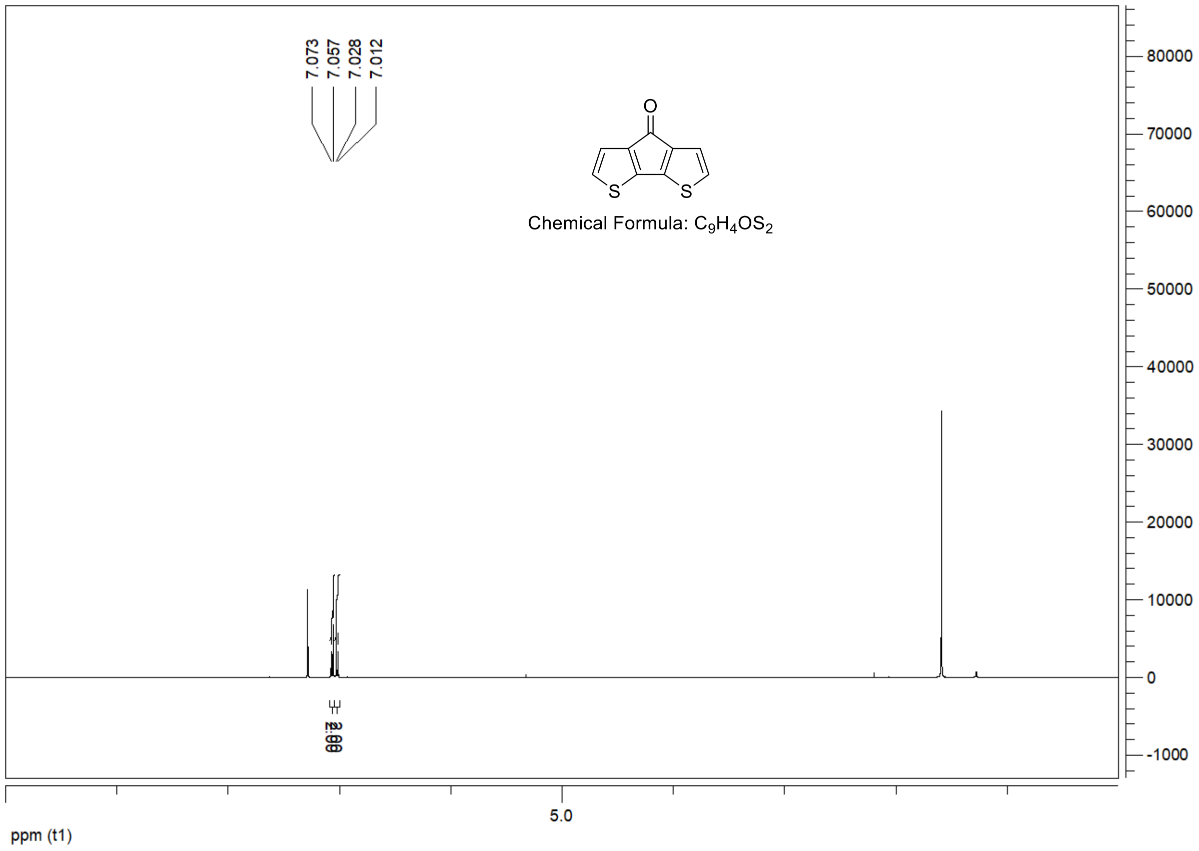

Suppl. Fig. 2: ^{1}H NMR data of 4H-cyclopenta[2,1-b:3,4-b']dithiophen-4-one (1)

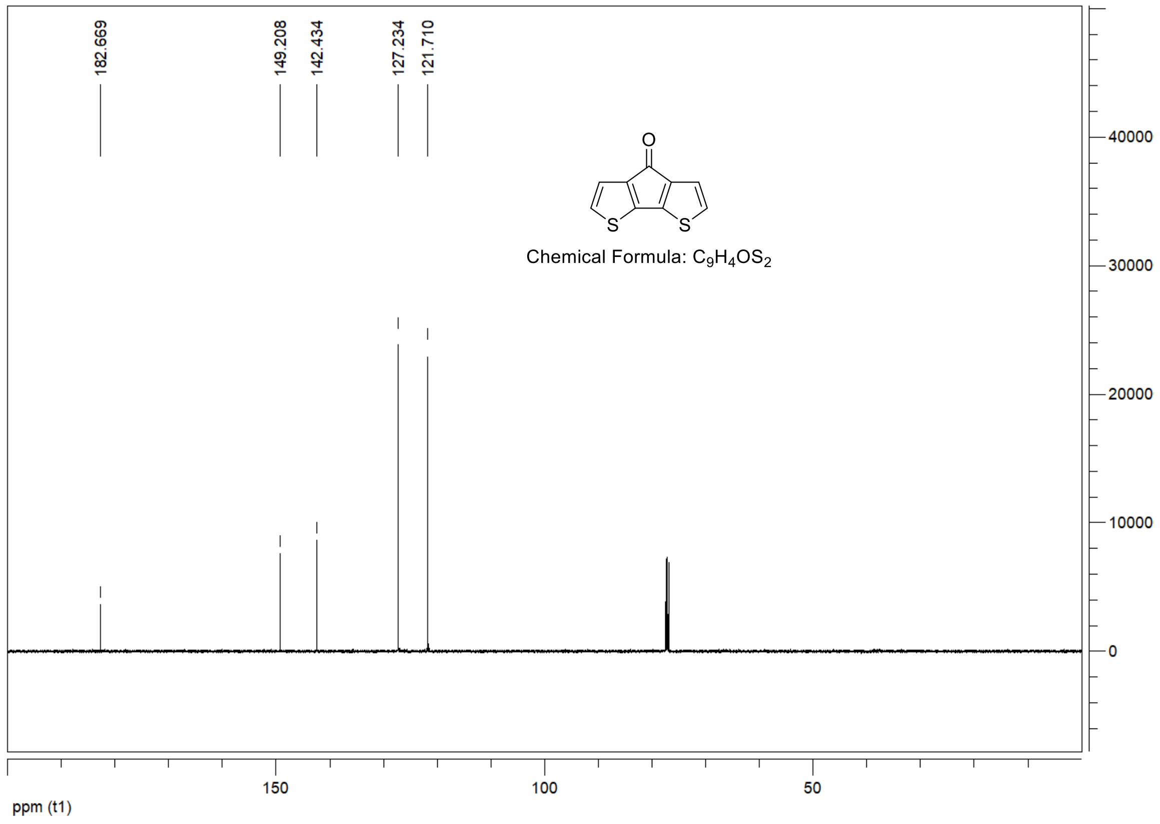

Suppl. Fig. 3: ^{13}C NMR data of 4H-cyclopenta[2,1-b:3,4-b']dithiophen-4-one (1)

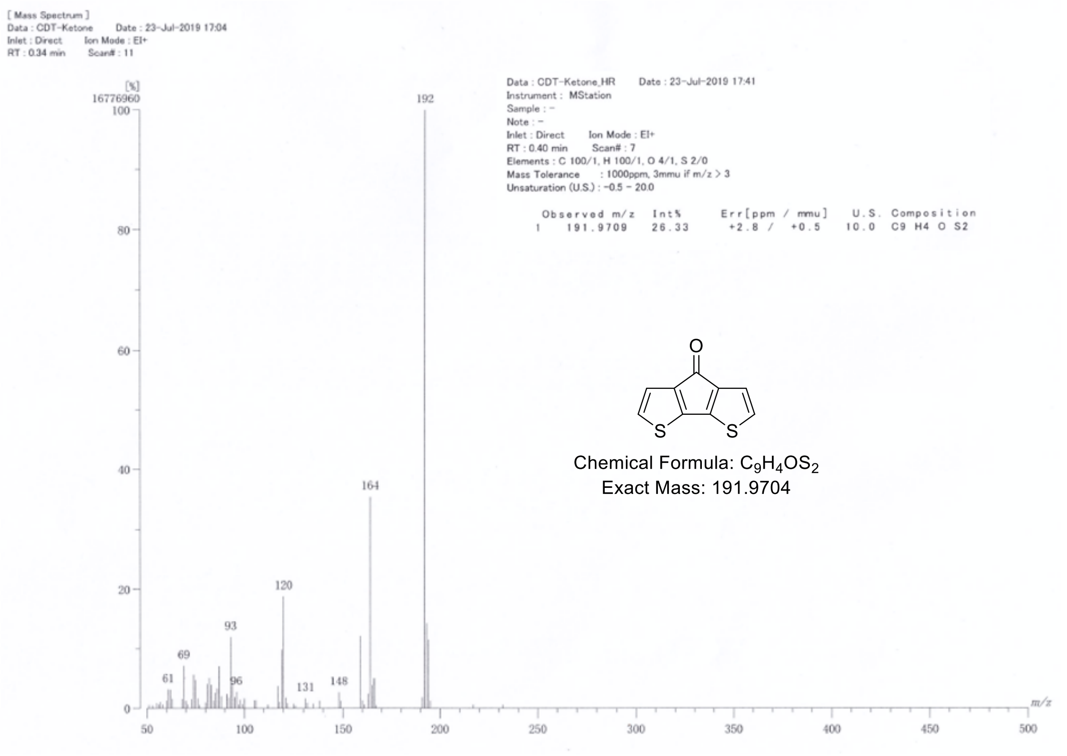

Suppl. Fig. 4: EI Mass data of 4H-cyclopenta[2,1-b:3,4-b']dithiophen-4-one (1)

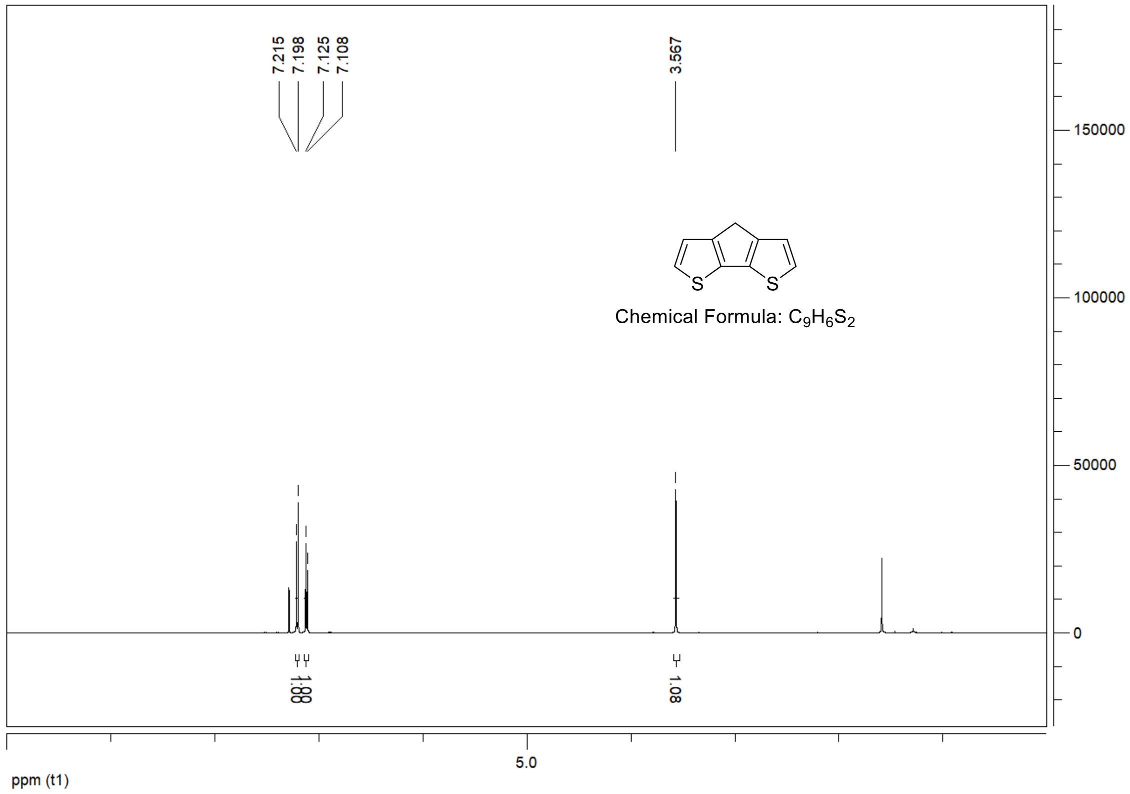

Suppl. Fig. 5: ^{1}H NMR data of 4H-cyclopenta[2,1-b:3,4-b']dithiophene (2)

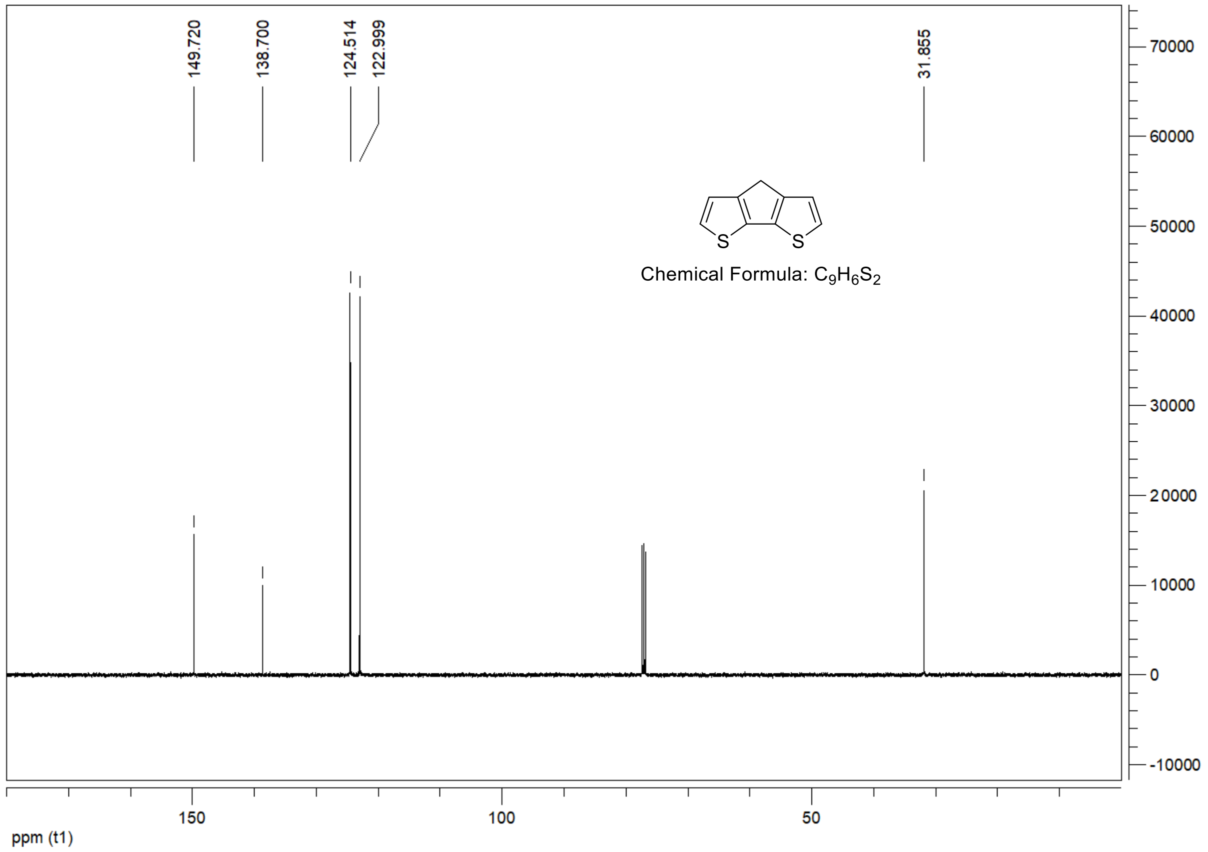

Suppl. Fig. 6: ^{13}C NMR data of 4H-cyclopenta[2,1-b:3,4-b']dithiophene (2)

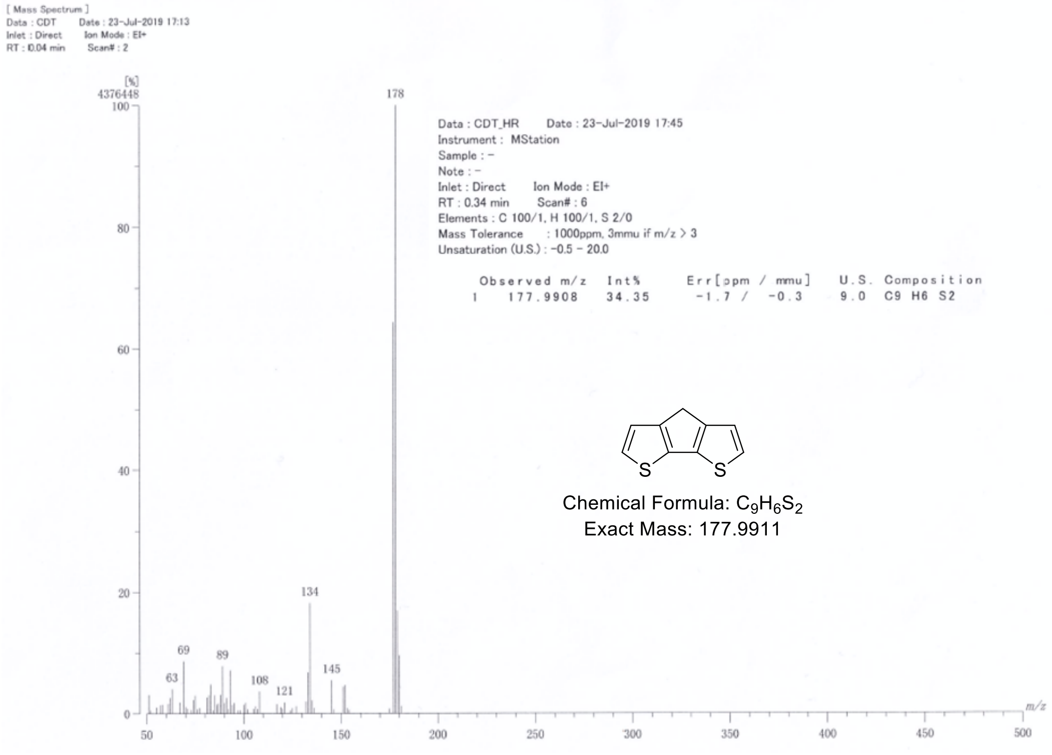

Suppl. Fig. 7: EI Mass data of 4H-cyclopenta[2,1-b:3,4-b']dithiophene (2)

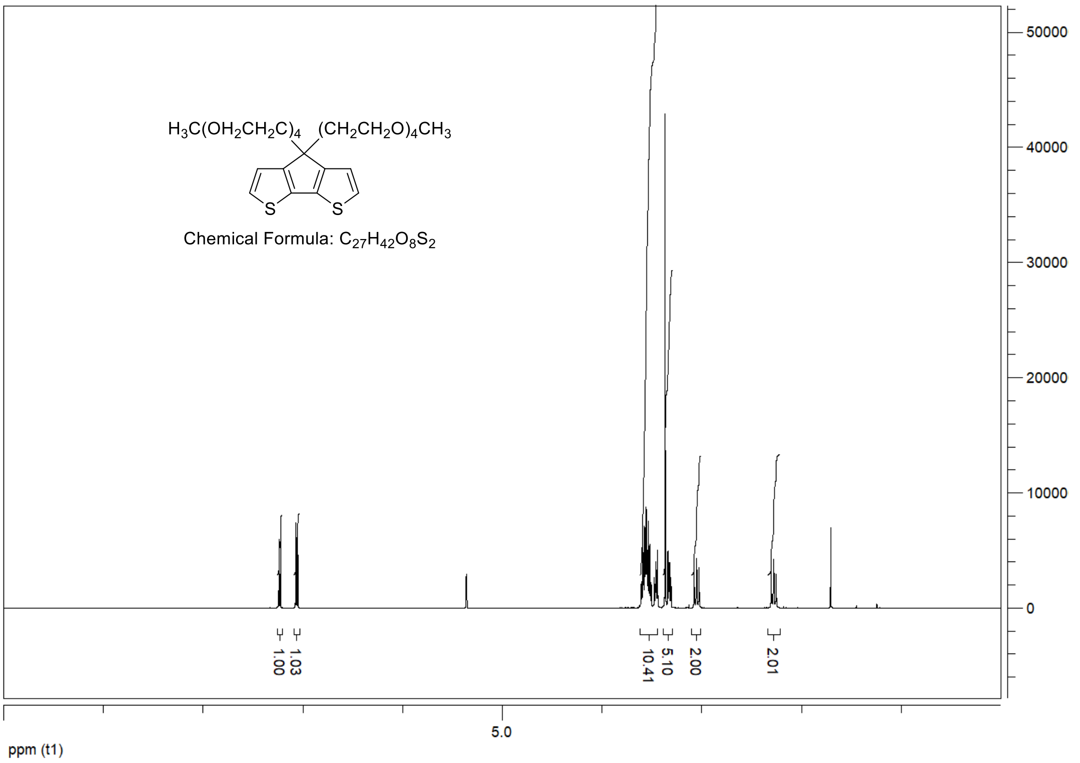

Suppl. Fig. 8: ^{1}H NMR data of 13,13'-(4H-cyclopenta[2,1-b:3,4-b']dithiophene-4,4-diyl)bis(2,5,8,11-tetraoxatridecane) (3)

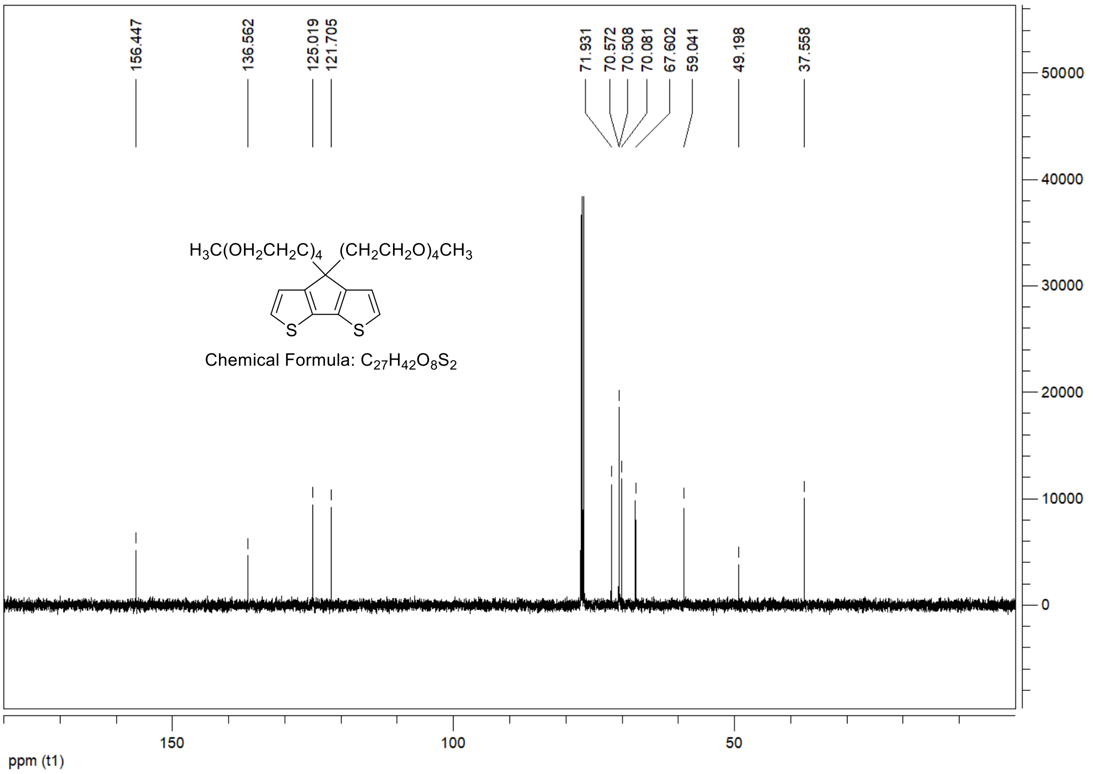

Suppl. Fig. 9: ^{13}C NMR data of 13,13'-(4H-cyclopenta[2,1-b:3,4-b']dithiophene-4,4-diyl)bis(2,5,8,11-tetraoxatridecane) (3)

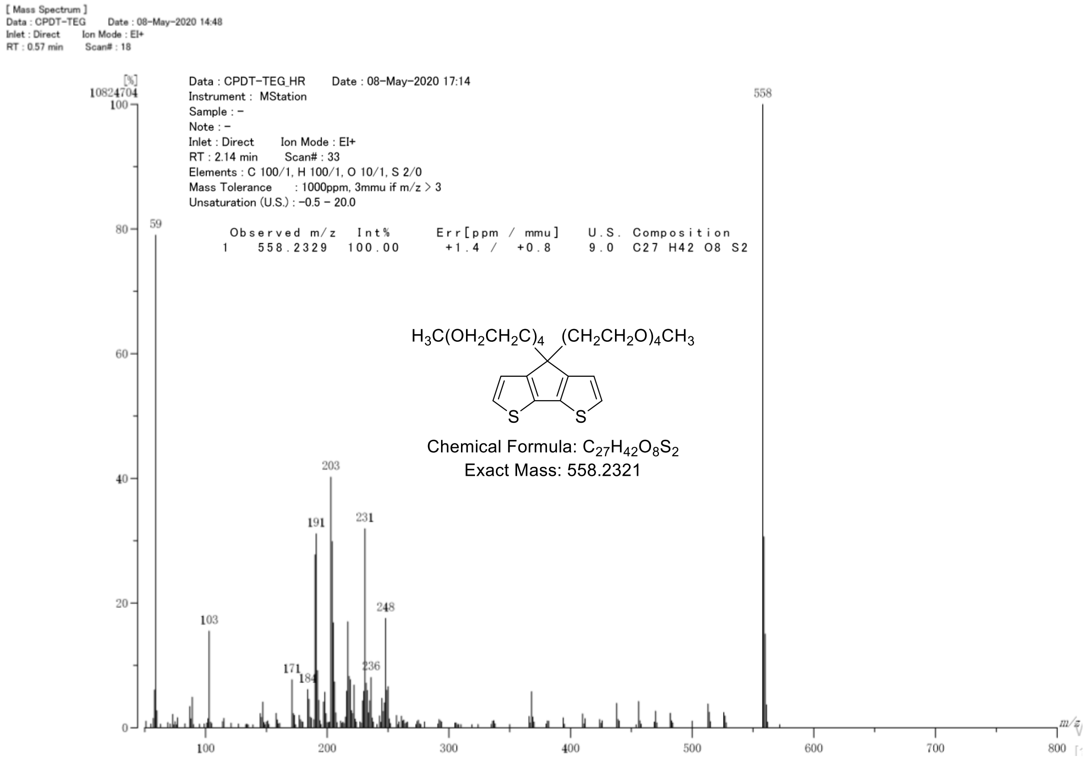

Suppl. Fig. 10: EI Mass data of 13,13'-(4H-cyclopenta[2,1-b:3,4-b']dithiophene-4,4-diyl)bis(2,5,8,11-tetraoxatridecane) (3)

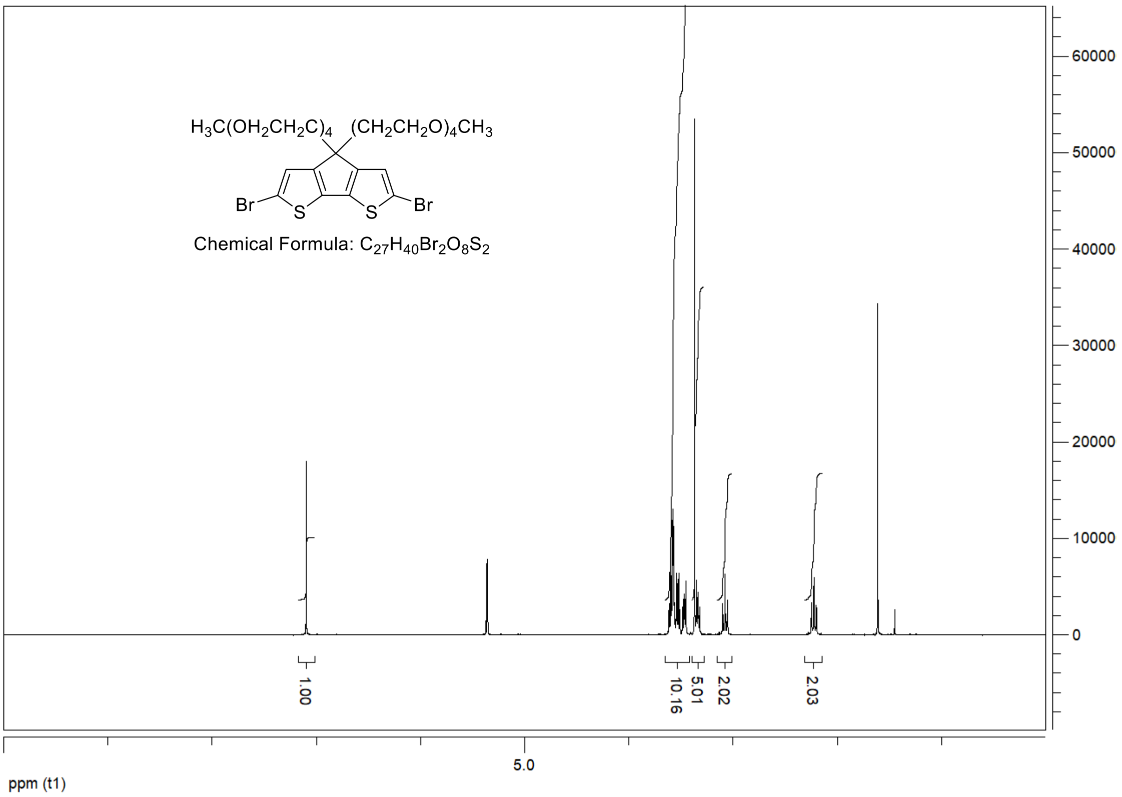

Suppl. Fig. 11: ^{1}H NMR data of 13,13'-(2,6-dibromo-4H-cyclopenta[2,1-b:3,4-b']dithiophene-4,4-diyl)bis(2,5,8,11-tetraoxatridecane) (4)

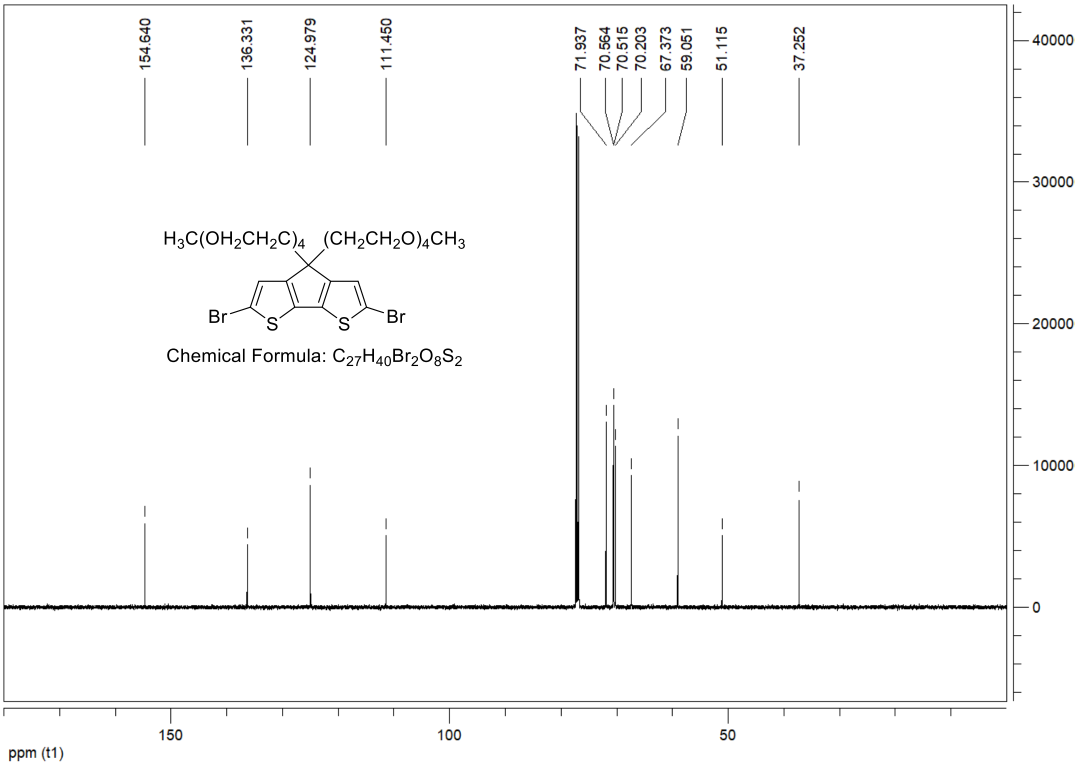

Suppl. Fig. 12: ^{13}C NMR data of 13,13'-(2,6-dibromo-4H-cyclopenta[2,1-b:3,4-b']dithiophene-4,4-diyl)bis(2,5,8,11-tetraoxatridecane) (4)

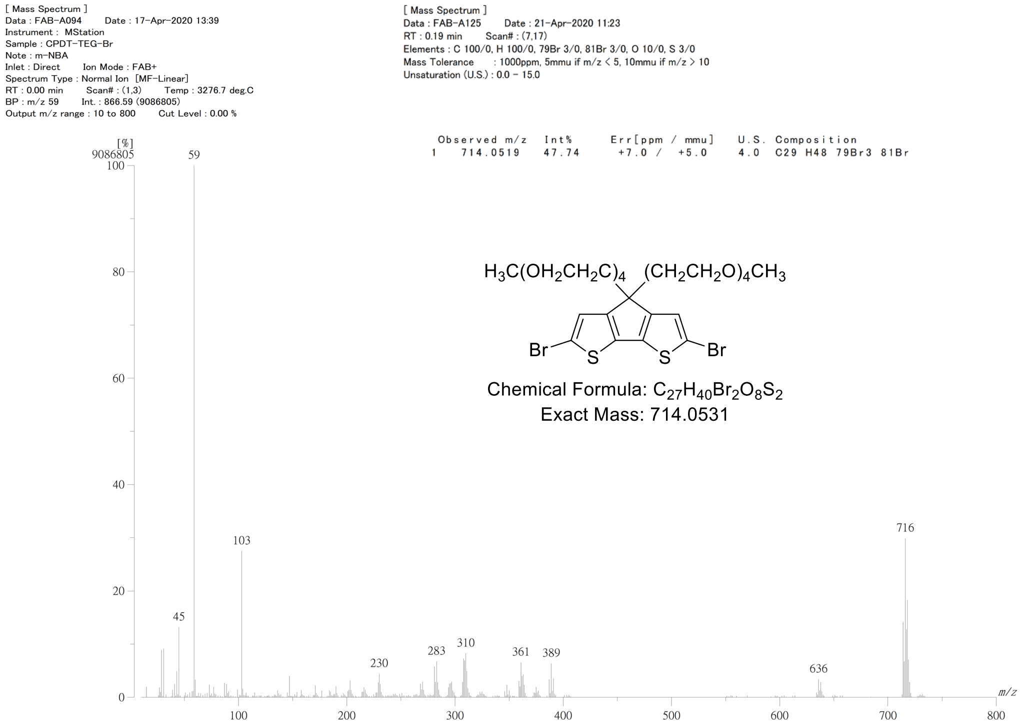

Suppl. Fig. 13: FAB Mass data of 13,13'-(2,6-dibromo-4H-cyclopenta[2,1-b:3,4-b']dithiophene-4,4-diyl)bis(2,5,8,11-tetraoxatridecane) (4)

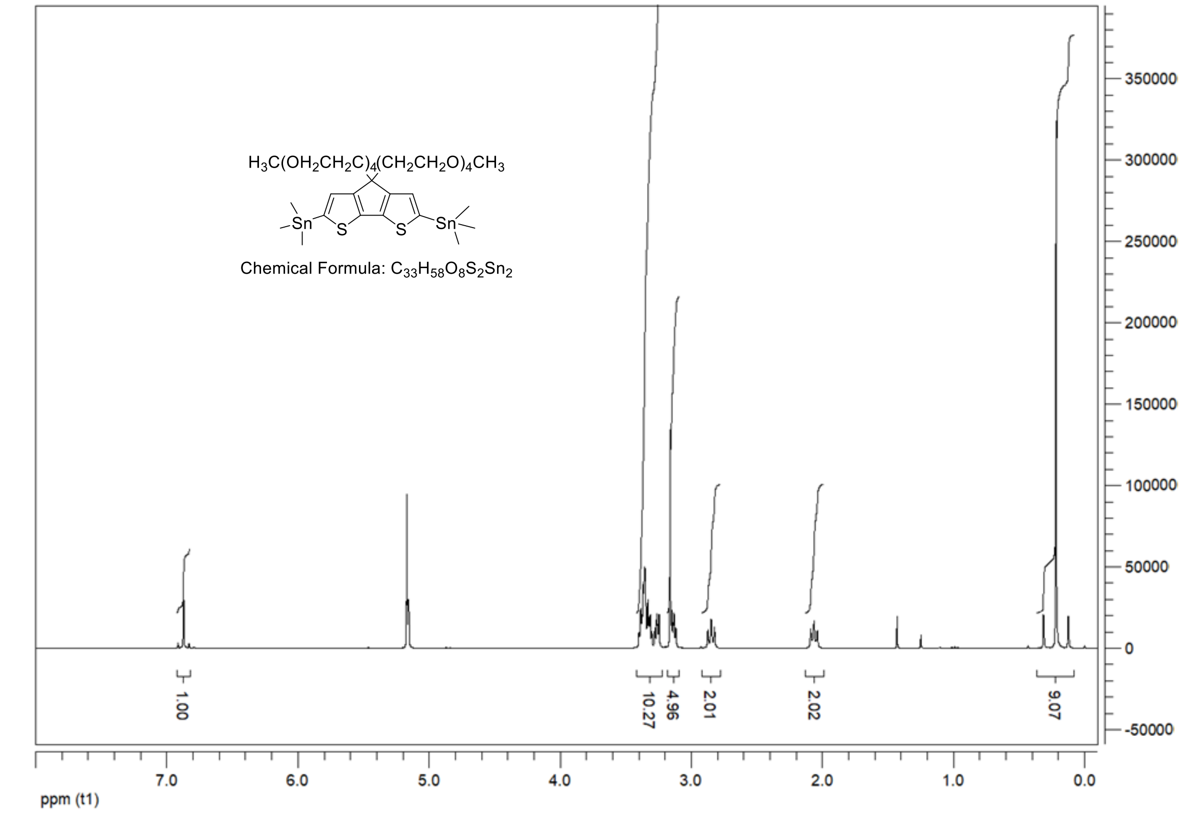

Suppl. Fig. 14: ^{1}H NMR data of (4,4-di(2,5,8,11-tetraoxatridecan-13-yl)-4H-cyclopenta[2,1-b:3,4-b']dithiophene-2,6-diyl)bis(trimethylstannane) (5)

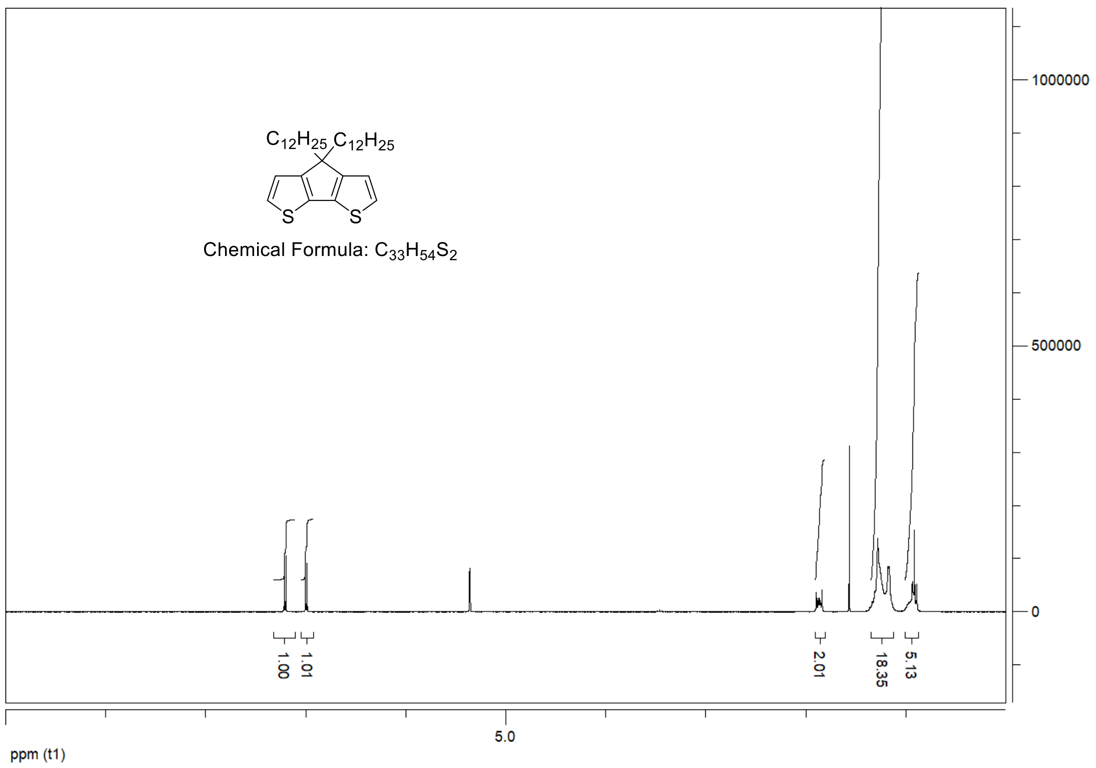

Suppl. Fig. 15: ^{1}H NMR data of 4,4-didodecyl-4H-cyclopenta[2,1-b:3,4-b']dithiophene (6)

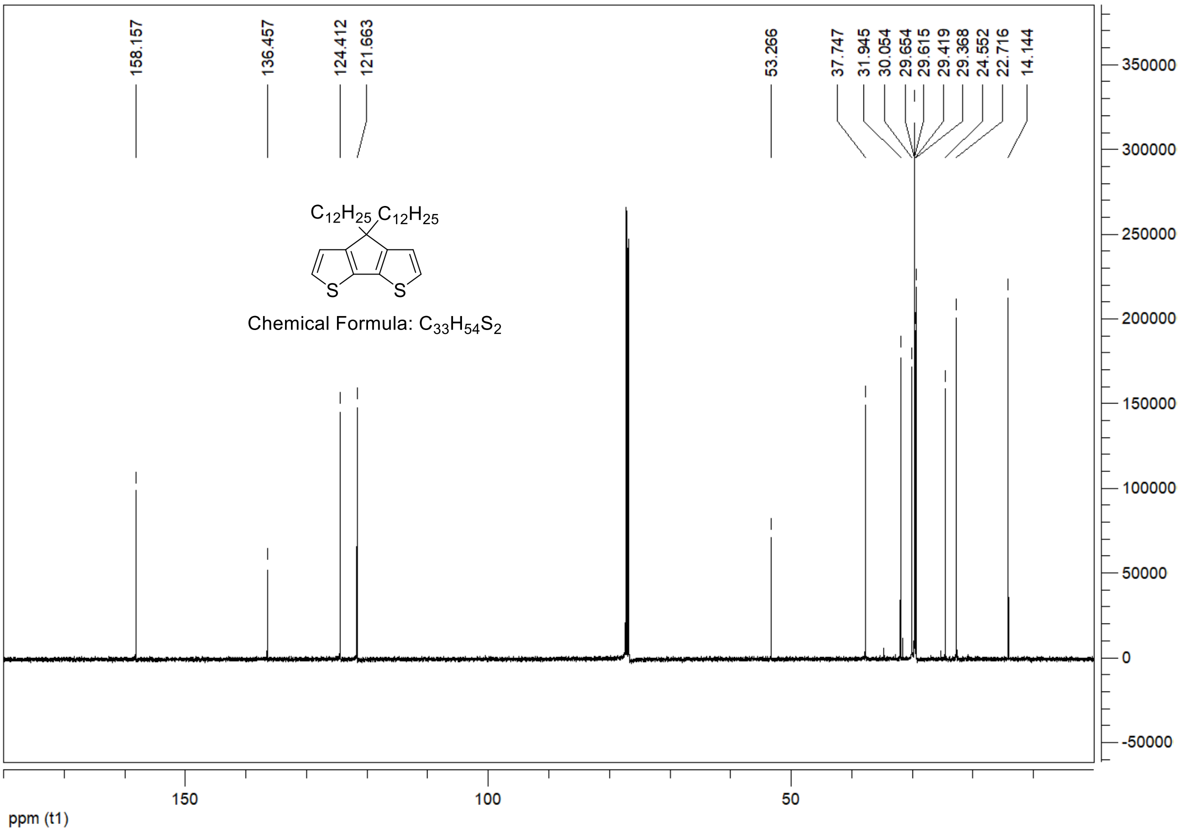

Suppl. Fig. 16: ^{13}C NMR data of 4,4-didodecyl-4H-cyclopenta[2,1-b:3,4-b']dithiophene (6)

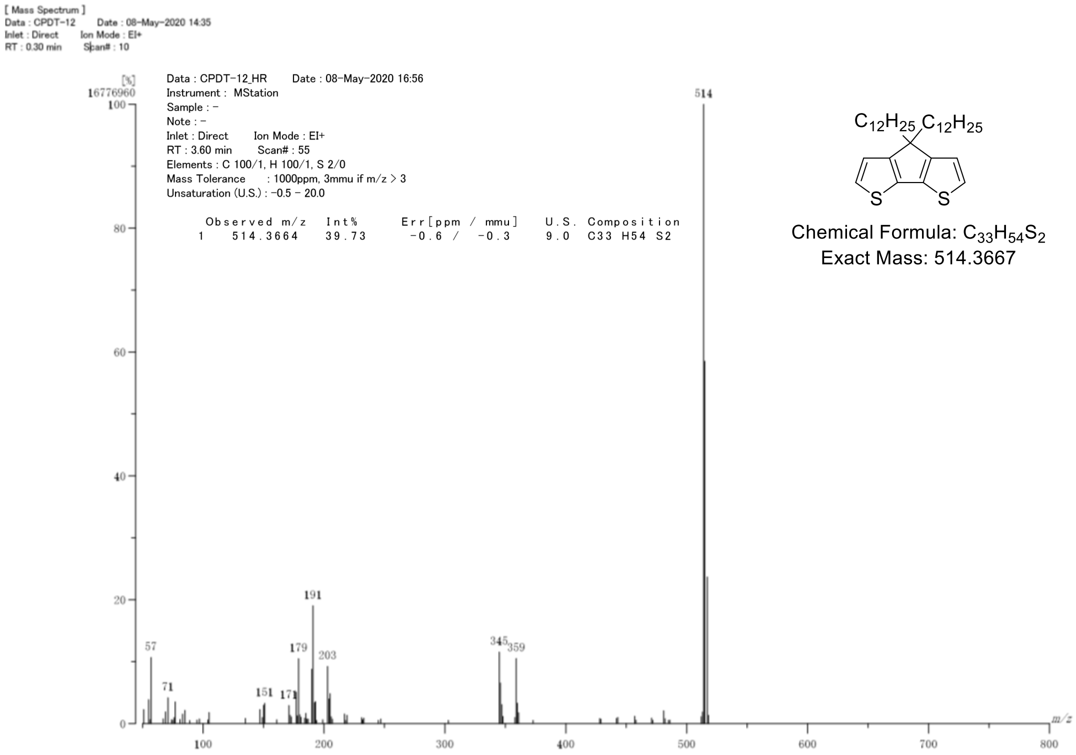

Suppl. Fig. 17: EI Mass data of 4,4-didodecyl-4H-cyclopenta[2,1-b:3,4-b']dithiophene (6)

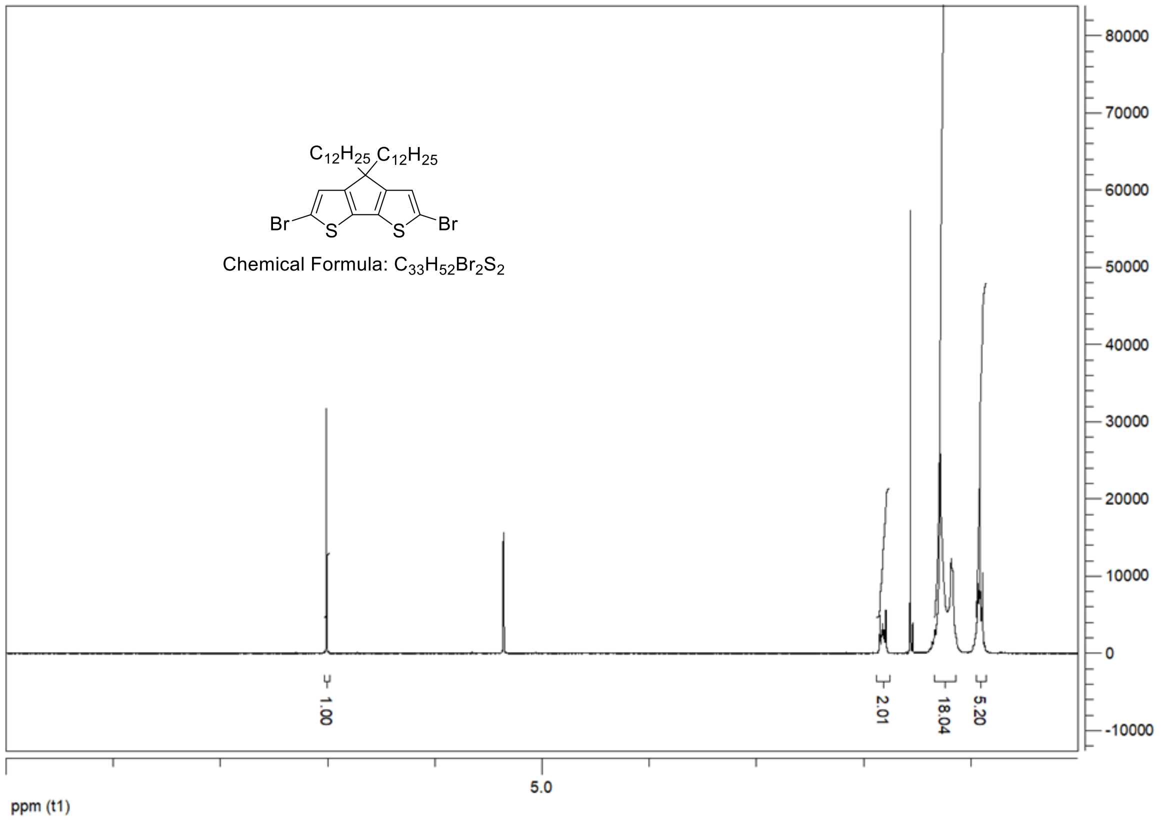

Suppl. Fig. 18: ^{1}H NMR data of 2,6-dibromo-4,4-didodecyl-4H-cyclopenta[2,1-b:3,4-b']dithiophene (7)

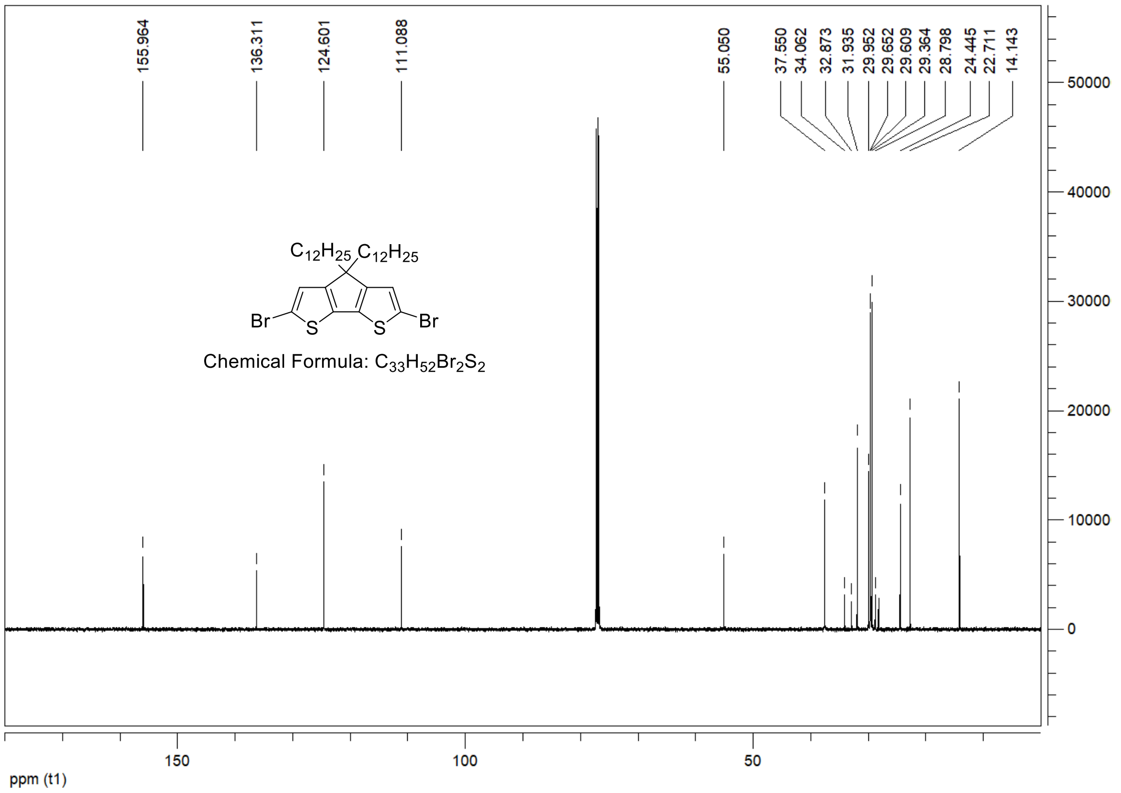

Suppl. Fig. 19: ^{13}C NMR data of 2,6-dibromo-4,4-didodecyl-4H-cyclopenta[2,1-b:3,4-b']dithiophene (7)

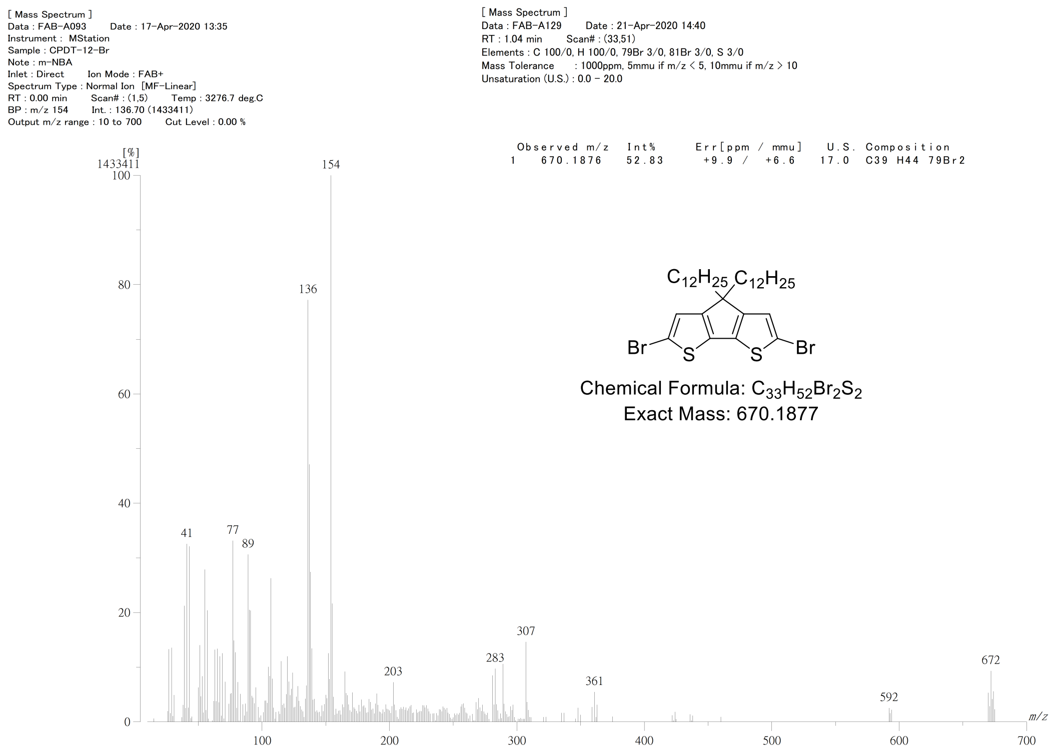

Suppl. Fig. 20: FAB Mass data of 2,6-dibromo-4,4-didodecyl-4H-cyclopenta[2,1-b:3,4-b']dithiophene (7)

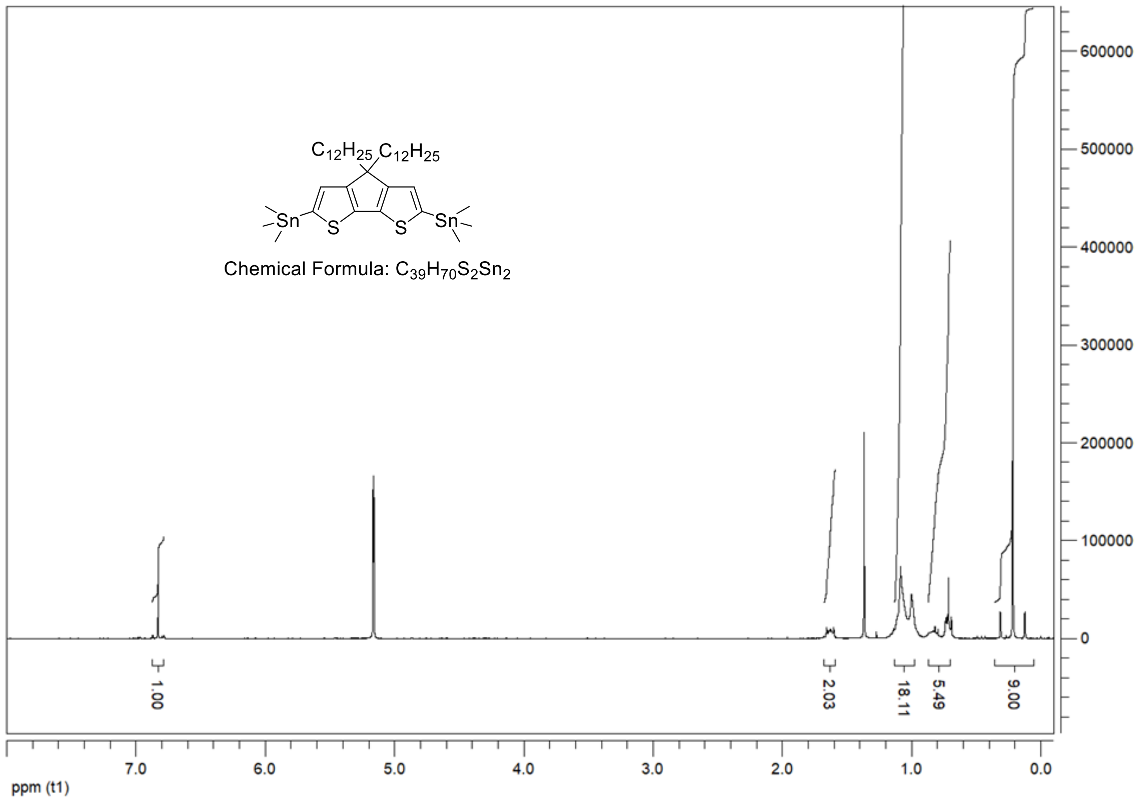

Suppl. Fig. 21: ^{1}H NMR data of (4,4-didodecyl-4H-cyclopenta[2,1-b:3,4-b']dithiophene-2,6-diyl)bis(trimethylstannane) (8)

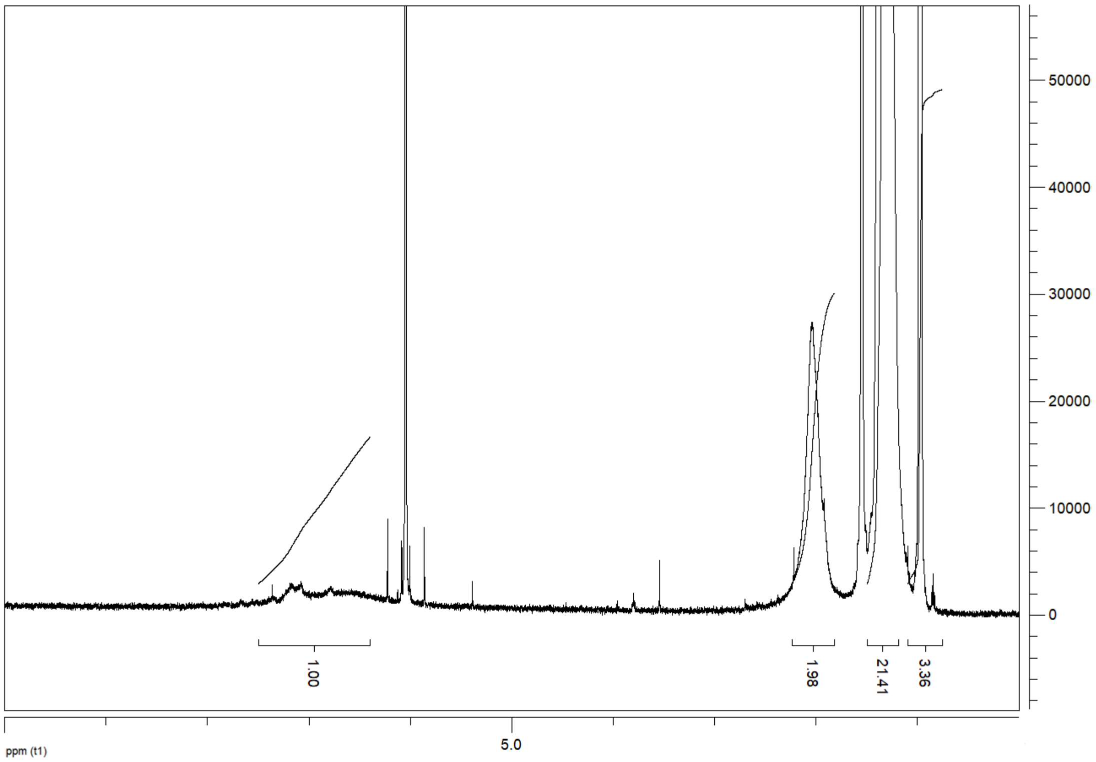

Suppl. Fig. 22: ^{1}H NMR data of CPDT g0%.

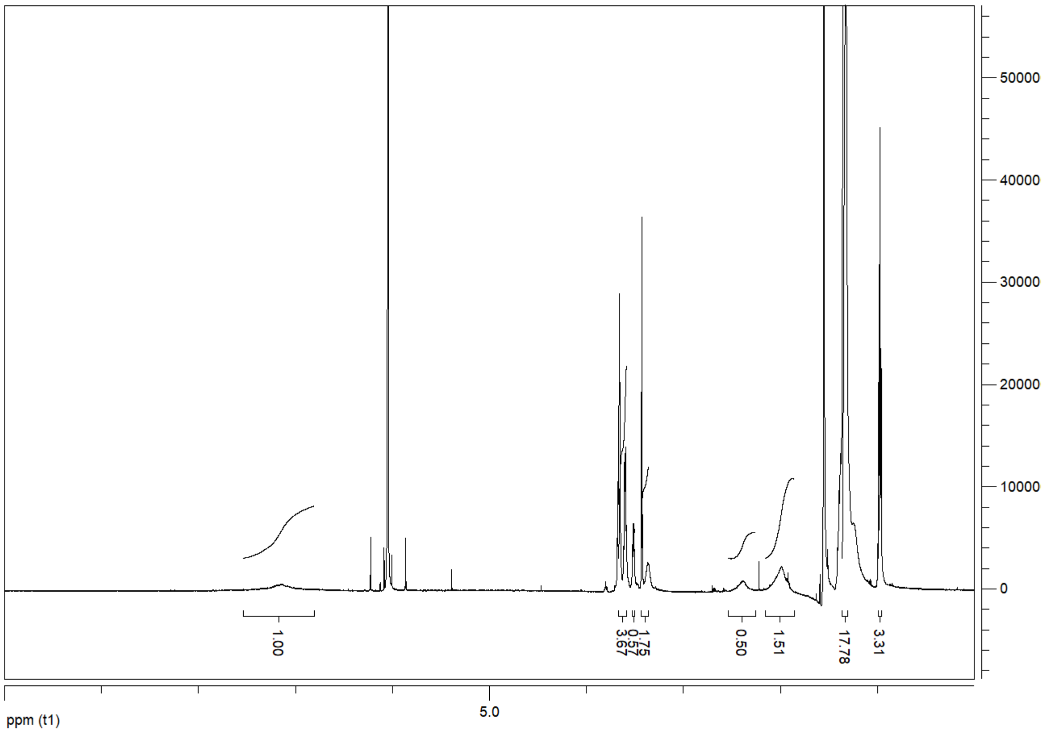

Suppl. Fig. 23: ^{1}H NMR data of CPDT g25%.

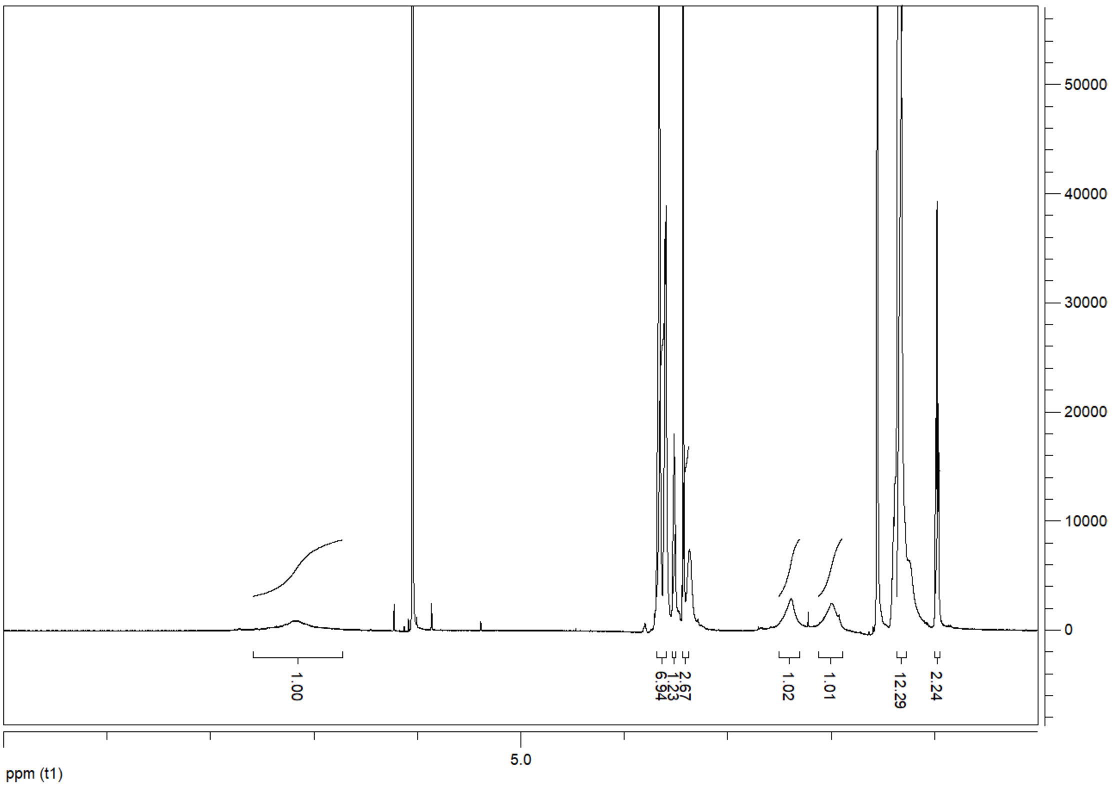

Suppl. Fig. 24: ^{1}H NMR data of CPDT g50%.

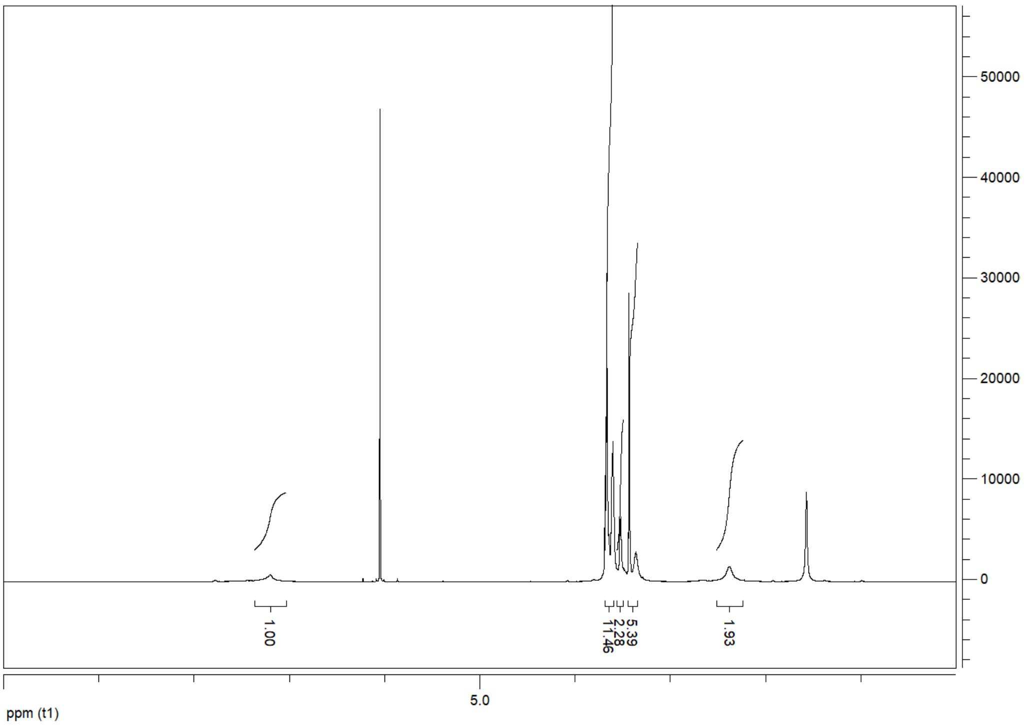

Suppl. Fig. 25: ^{1}H NMR data of CPDT g100%

Supplementary Note 1: Material properties

Suppl. Table 1 summarises the molecular numbers, molecular weights and PDIs of the polymers. Solution and film UV-Vis-NIR absorption spectra were recorded by using a Cary 5000 UV-Vis-near IR double-beam spectrophotometer and are shown in Suppl. Fig. 26. The CV scans of the CPDT polymers are shown in Suppl. Fig. 27. Suppl. Fig. 28 shows the thermogravimetry analysis (TGA) and differential scanning calorimetry (DSC) curves of the CPDT polymers. As of glycol sidechain content increases, T_d and T_m gradually decrease while crystallinity distinctly appears with T_c and T_m.

Suppl. Table 1: Polymer properties. Summary of the *M*n and *M*w values for the CPDT polymer series, measured with GPC at 40°C in chloroform.

Polymer	*M*n (kg mol^{-1})	*M*w (kg mol^{-1})	Đ
g0%	22	49	2.2
g25%	22	50	2.3
g50%	23	55	2.4
g100%	20	66	3.4

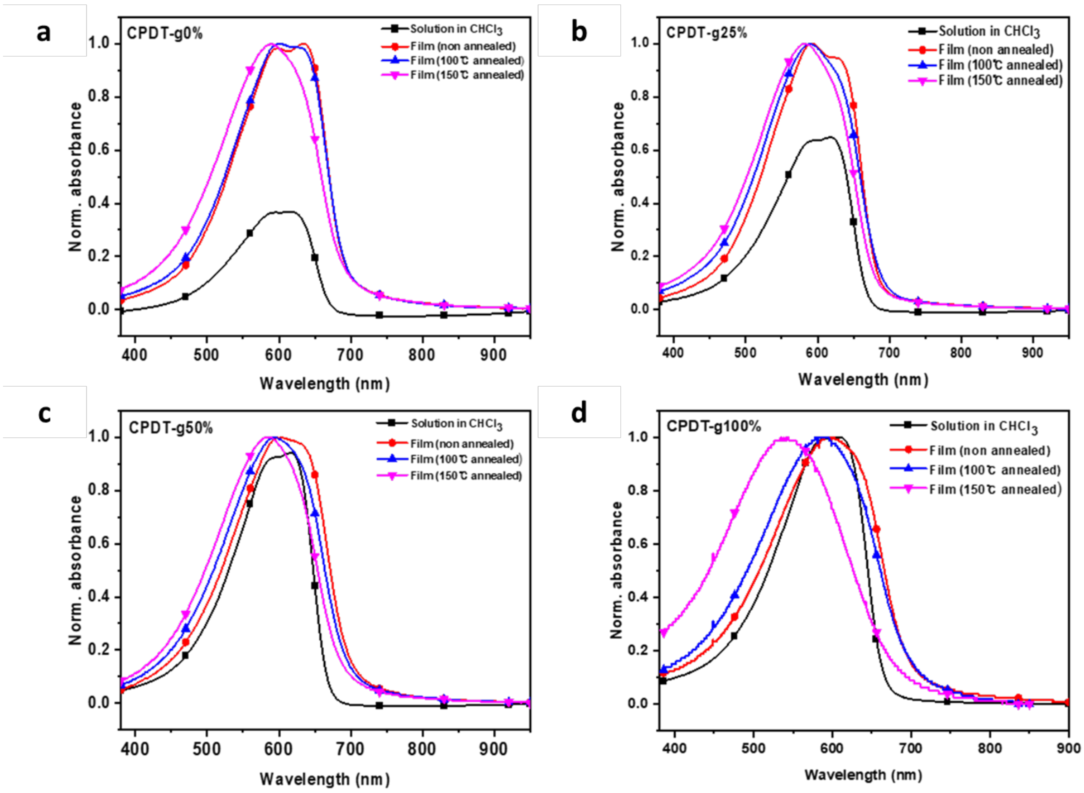

Suppl. Fig. 26: Solution and thin-film UV-vis absorption spectra of the CPDT polymers. a CPDT-g0%, **b** CPDT-g25%, **c** CPDT-g50%, **d** CPDT-g100%. For each polymer, UV-Vis spectra are shown in $CHCl_3$ solution

(black squares), non-annealed thin films (red circles), thin films annealed at 100°C (blue triangles) and thin films annealed at 150°C (pink nablas).

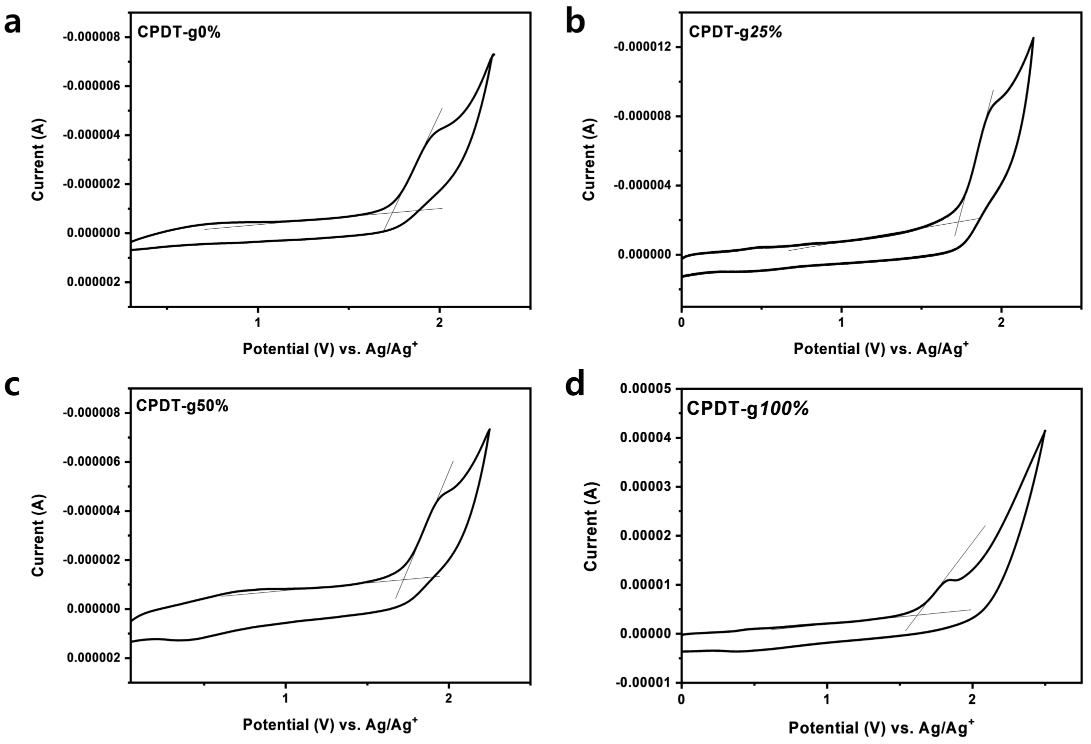

Suppl. Fig. 27: Cyclic voltammogram (CV) curves of the CPDT polymers. **a** CPDT-g0%, **b** CPDT-g25%, **c** CPDT-g50%, **d** CPDT-g100%.

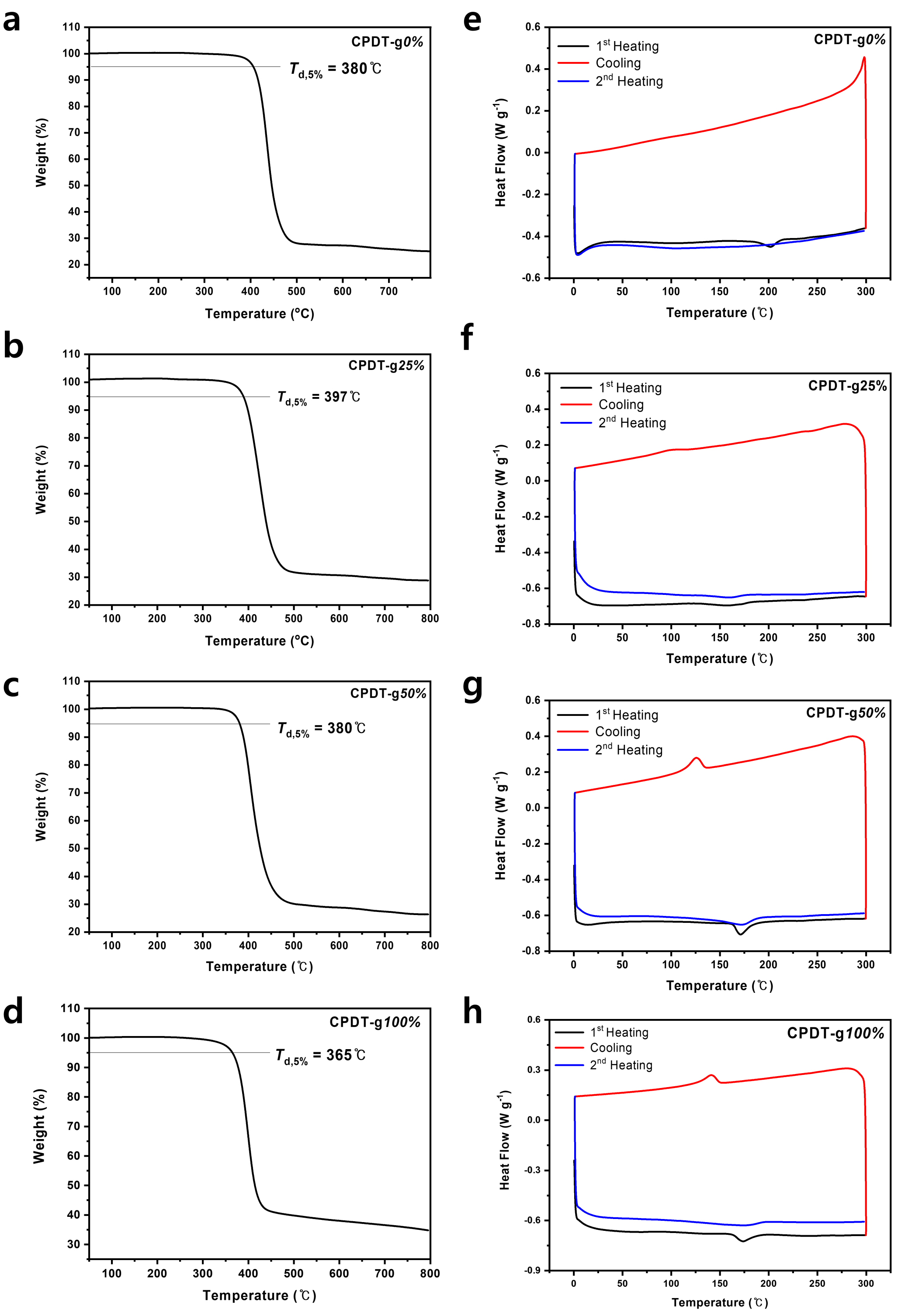

Suppl. Fig. 28: Thermogravimetry Analysis and differential scanning calorimetry. Thermogravimetry Analysis (TGA) (**a-d**) and differential scanning calorimetry (DSC) curves of the CPDT polymers (**e-h**).

Suppl. Table 2: General polymer properties. Thermal, optical and electrochemical properties of the CPDT polymers.

Compound	T_d [a)] (°C)	T_m (°C)	T_c (°C)	UV-λ_{max} [b)] (nm)	E_g [c)] (eV)	HOMO [d)] (eV)	LUMO [e)] (eV)
CPDT-g0%	406	202	-	617	1.75	-6.21	-4.46
CPDT-g25%	397	188	-	616	1.78	-6.19	-4.41
CPDT-g50%	380	171	125	615	1.73	-6.18	-4.45
CPDT-g100%	365	173	141	609	1.70	-6.00	-4.30

[a)] The temperature of 5% weight-loss under nitrogen.

[b)] Chloroform solution at 298 K.

[c)] Calculated from the absorption edge, Eg=1240/λabs, onset.

[d)] HOMO level was calculated from oxidation onset.

[e)] LUMO level was obtained from HOMO and Eg.

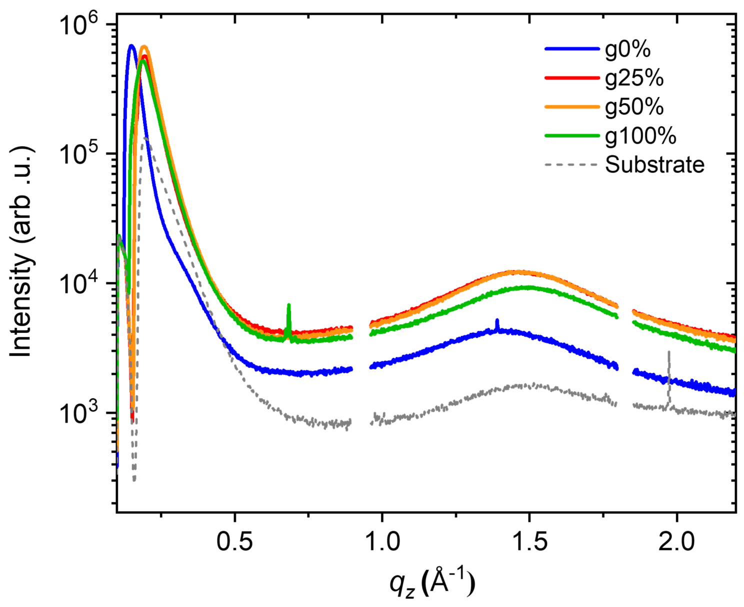

Suppl. Fig. 29: Out-of-plane GIWAXS. The out-of-plane (q_z) scattering profile for the CPDT polymer series.

Suppl. Table 3: *d*-spacing values from GIWAXS. Scattering peaks and *d*-spacing values extracted from 2D GIWAXS diffractograms of the CPDT polymer series.

	In-plane				Out-of-plane			
	Lamellar stacking		π-stacking		Lamellar stacking		π-stacking	
**Sample**	**q_{xy}^{100}(Å^{-1})**	***d*(Å)**	**q_{xy}^{π}(Å^{-1})**	***d*(Å)**	**q_z^{100}(Å^{-1})**	***d*(Å)**	**q_z^{π}(Å^{-1})**	***d*(Å)**
0	0.27	23.02	1.36	4.62	0.15	42.12	1.44	4.36
25	0.28	22.85	1.43	4.40	0.19	32.55	1.48	4.24
50	0.27	23.02	1.44	4.37	0.19	32.55	1.46	4.30
100	0.23	26.97	1.46	4.32	0.19	33.18	1.47	4.27
Substrate	-	-	1.52	4.12	0.19	32.55	1.45	4.34

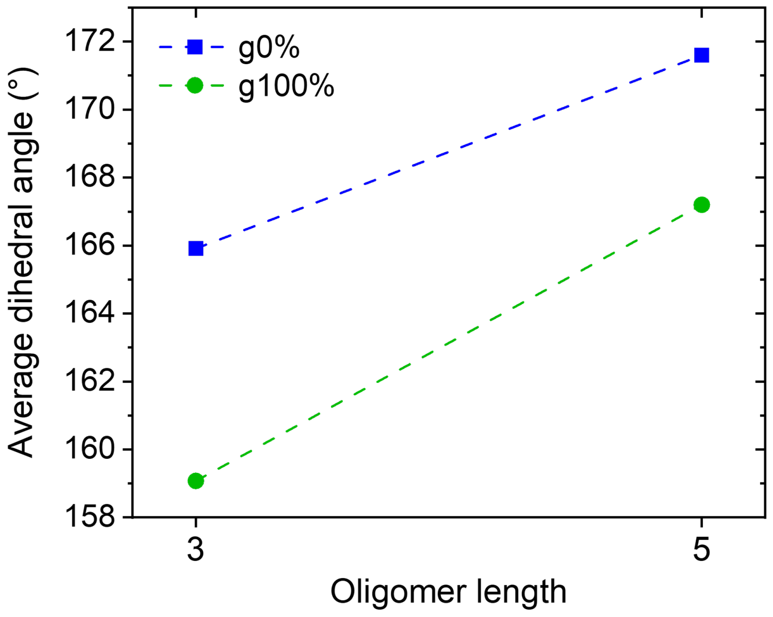

Suppl. Fig. 30: Simulated ground-state dihedral angles. Average dihedral angles of ground state trimers and pentamers of g0% (blue squares) and g100% (green circles) oligomers simulated in DFT.

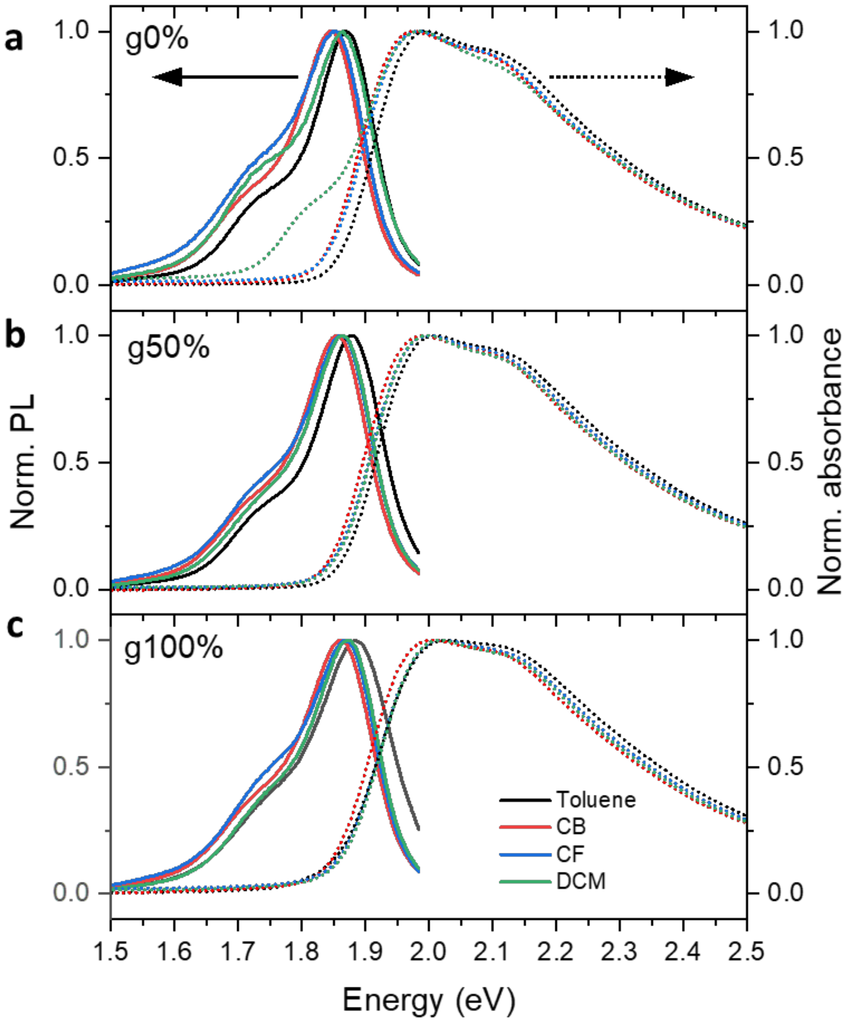

Suppl. Fig. 31: Solvatochromic data. Normalised absorbance and PL spectra (in photons per energy interval) of the **a** g0%, **b** g50% and **c** g100% polymers in solution. Spectra were taken in toluene, chlorobenzene (CB), chloroform (CF) and dichloromethane (DCM), and polymers were at a concentration of 0.01 mg/ml. A shoulder of emission can be seen in the g0% DCM absorption spectrum, which likely arises due to the poor solubility of the alkylated polymers in the polar solvent.

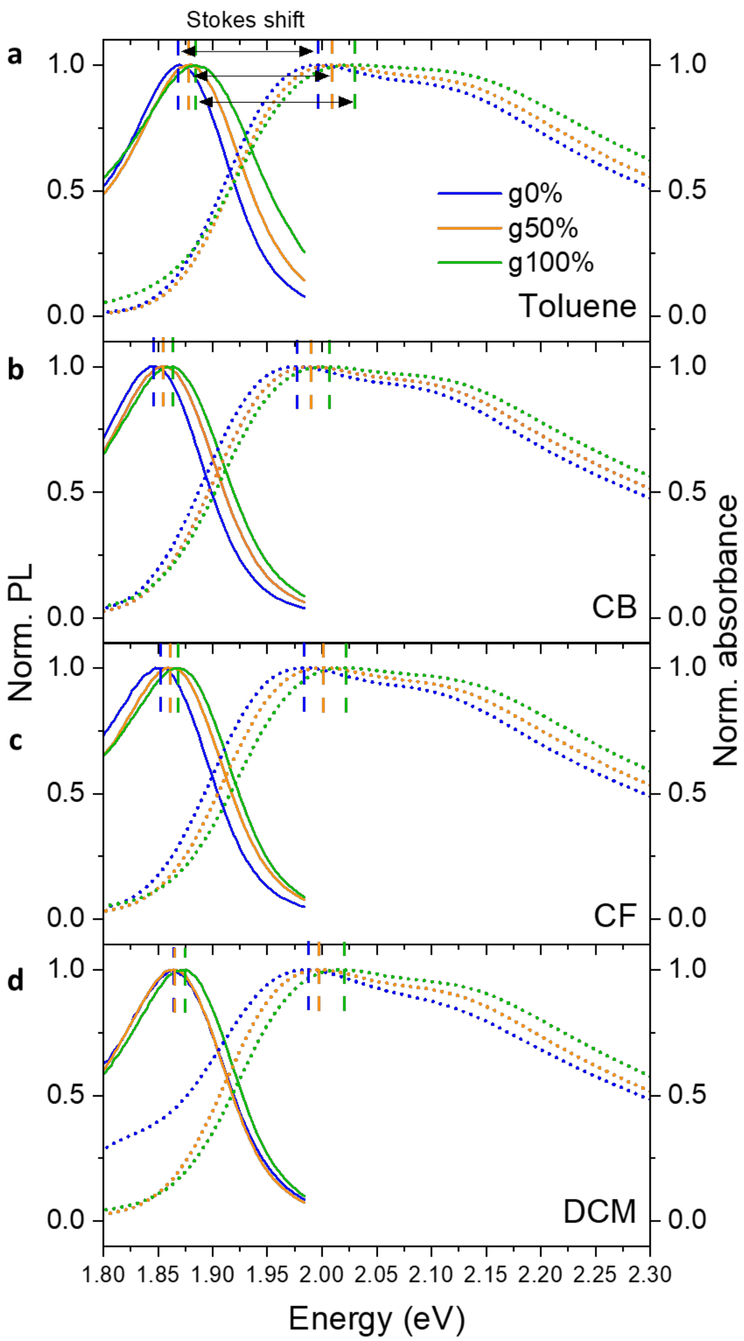

Suppl. Fig. 32: Method of Stokes shift extraction. Method of Stokes shift extraction for the g0%, g50% and g100% polymers in solutions of **a** toluene, **b** CB, **c** CF and **d** DCM. The Stokes shift is taken as the energy difference between the maxima of the absorbance and PL spectra, which are indicated with vertical dashed lines for each spectrum. An example of Stokes shift extractions is shown for the toluene spectra in panel **a**.

Suppl. Table 4: Absorbance and PL spectra maxima. Maxima positions of the absorbance and PL spectra for each polymer and the associated Stokes shifts.

Polymer	Solvent	Absorbance maximum (eV)	PL maximum (eV)	Stokes shift (eV)
g0%	Toluene	1.996	1.870	0.126
	CB	1.977	1.845	0.132
	CF	1.984	1.853	0.131
	DCM	1.987	1.864	0.123
g50%	Toluene	2.009	1.881	0.128
	CB	1.990	1.853	0.137
	CF	2.000	1.859	0.141
	DCM	1.996	1.864	0.130
g100%	Toluene	2.029	1.884	0.145
	CB	2.006	1.864	0.142
	CF	2.019	1.867	0.152
	DCM	2.019	1.876	0.144

›

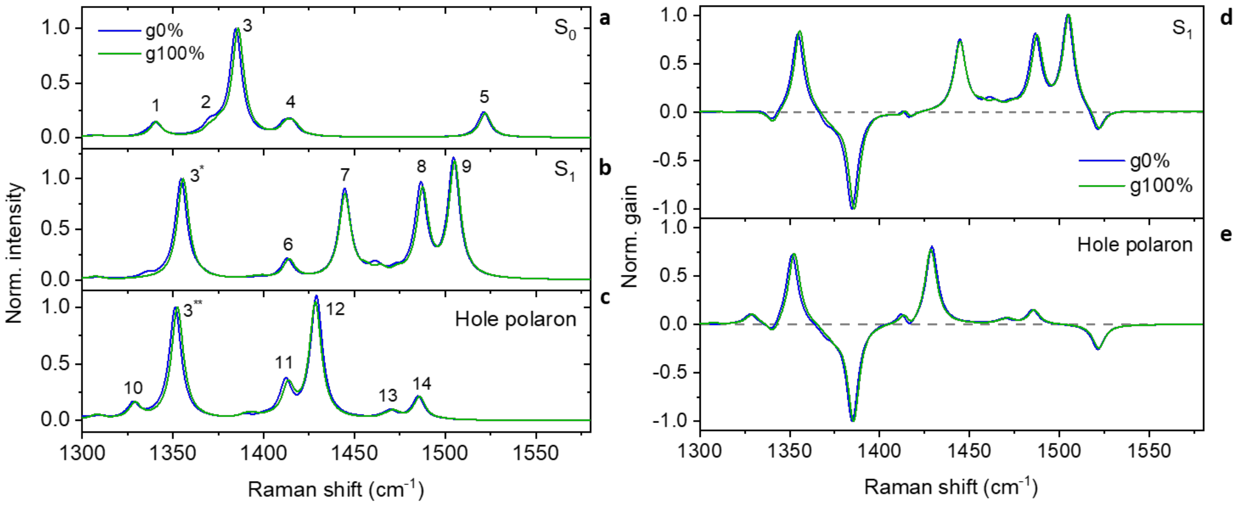

Suppl. Fig. 33: Simulated Raman spectra. DFT-simulated Raman spectra of isolated g0% and g100% trimers in **a** the ground state, S_0, **b** the excited state, S_1 and **c** for a hole polaron. **d** Raman gain spectra for the g0% and g100% trimers in the S_1 state and **e** hole polaron state. The spectrum is obtained by deducting the S_1 Raman spectrum from the S_0 Raman spectrum.

Suppl. Table 5: Simulated Raman spectra assignments. Vibrational mode assignments for the DFT-simulated g0% and g100% Raman spectra in Suppl. Fig. 33. The asterisks indicate the shifted peak frequency in the S_1 state (*) and hole polaron state (**).

Peak	Raman shift (cm^{-1})	Assignment
1	1340	C=C entire backbone
2	1370	C-C intra-ring cyclopentane
3	1384, 1355*, 1350**	C-C intra-ring cyclopentane, some C=C entire backbone
4	1414	C-C intra thiophene
5	1522	Outer C=C mode on thiophene rings, neighbouring C-C inter ring
6	1413	C-C intra ring
7	1445	C-C intra ring cyclopentane
8	1487	C-C intra ring thiophene
9	1505	C-C inter ring
10	1327	C=C entire backbone
11	1412	C-C intra
12	1430	C-C intra
13	1470	C-C intra (central unit), C=C thiophenes (outer units)
14	1485	C-C inter ring

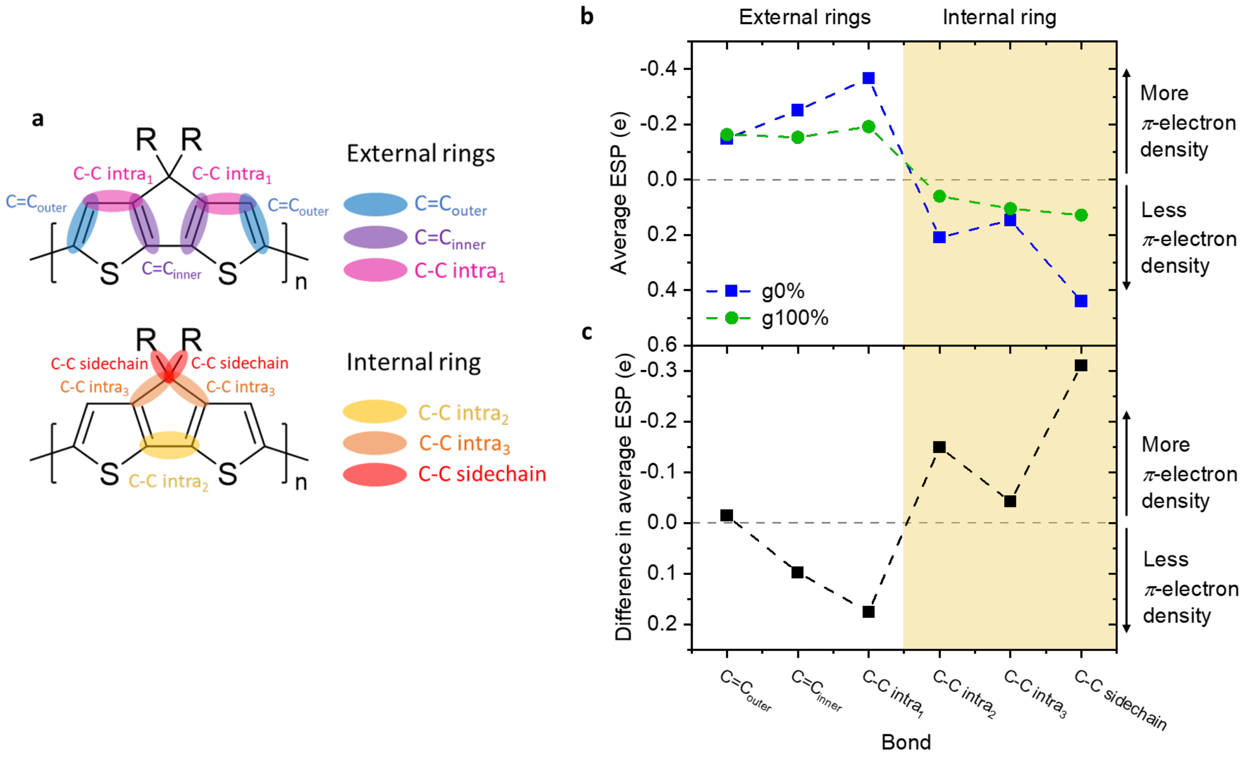

Suppl. Fig. 34: ESP calculations. a Bond labels for the g0% and g100% oligomers. **b** Average ESP for g0% and g100% trimers for each bond along the backbone and C-C sidechain, being the first carbon-carbon bond in the sidechain. **c** Difference in average ESP between the g0% and g100% trimers, showing the impact of full glycolation on the ESP along the backbone.

Supplementary Note 2: ESP calculations

To support the conclusions made experimentally regarding π-electron density being more localised to the cyclopentane ring in the ground state g100% polymer backbone, we carried out further DFT calculations to simulate the electrostatic potential distribution (ESP) along the backbone of the ground state g0% and g100% trimers. This provides further insight into the impact of the glycol sidechains on the intrinsic, intramolecular π-electron distribution along the conjugated backbone. The ESPs of the g0% and g100% trimers were simulated according to the Merz-Kollman (MK) scheme and ESP was then calculated for each bond along the backbone and averaged according to bond type across the trimers. Suppl. Fig. 34 shows the average ESP for each bond along the backbone for the g0% and g100% trimers in the ground state and the change in the average ESP for each bond upon glycolation of the trimer. The results show that compared to the alkylated trimer, the glycolated trimer has less π-electron density localised to the outer thiophene ring C=C$_{inner}$ and C-C intra$_1$ bonds and more π-electron density localised to the central cyclopentane ring C-C intra$_2$ and C-C sidechain bonds. So, the g100% trimer has greater π-electron density concentration in the internal ring of the CPDT unit. This is consistent with the experimental results shown in Figure 3 and supports the conclusion of a more localised π-electron density in the g100% polymer.

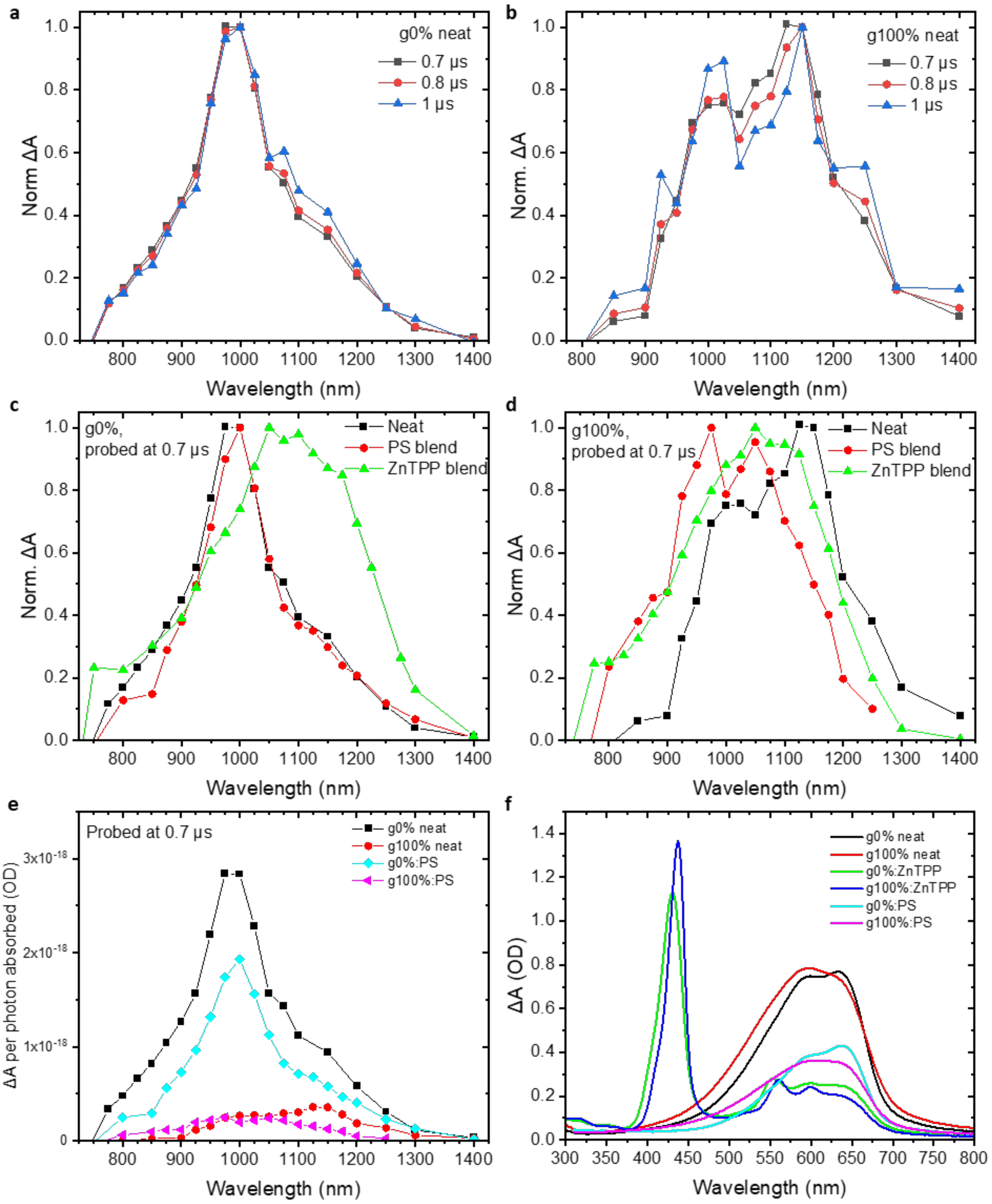

Suppl. Fig. 35: Microsecond TAS. Normalised μs-TA spectra of **a** g0% neat polymer and **b** g100% neat polymer films at various pump-probe delays, probed at 700 nm – 1400 nm, after photoexcitation at 600 nm, 12μJ cm^{-2}. Both the g0% and g100% spectra have two different bands with different lifetimes, centred at ~980 nm and ~1150 nm, assigned to the charge and triplet, respectively. Normalised TA spectra of the **c** g0% neat and **d** g100%, 1:1 polystyrene (PS) blend and 1:1 zinc tetraphenylporphyrin (ZnTPP) blend films, probed at 0.7μs. Neat and PS blend films were excited at 600 nm, 12μJ cm^{-2}, and ZnTPP blend films were excited at 430 nm, 12μJ cm^{-2}. The sensitised g0% triplet shows a broad band ~1100 nm, clearly further to the red than the sharp 980 nm peak observed for the pristine and PS blend film. The 980 nm band can thus be assigned to charges, consistent with the identical spectral position of the polaron pairs in the ps-TAS. **e** TA spectra of the g0% and g100% neat and PS blend films, corrected for number of photons absorbed, showing that the g0% polymer possesses a greater charge population than g100% at 0.7 μs. **f** Ground state absorbance of the g0% and g100% neat and blend films.

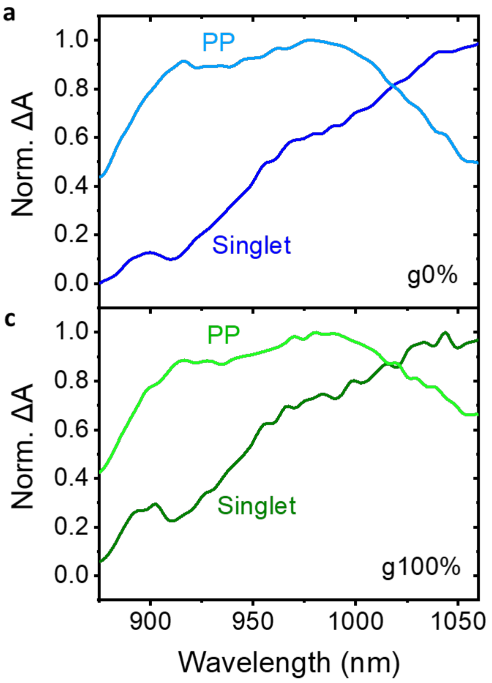

Suppl. Fig. 36: TAS spectra and kinetics. Deconvoluted TA spectra [**a** and **c**] obtained using global analysis and their corresponding kinetics [**b** and **d**] normalised to the singlet signal maximum. It shows that in both polymers singlet excitons form upon photoexcitation and undergo ultrafast polaron pair (PP) formation. Singlet spectra are fit with a biexponential decay and PP signals are fit with a multiexponential fit.

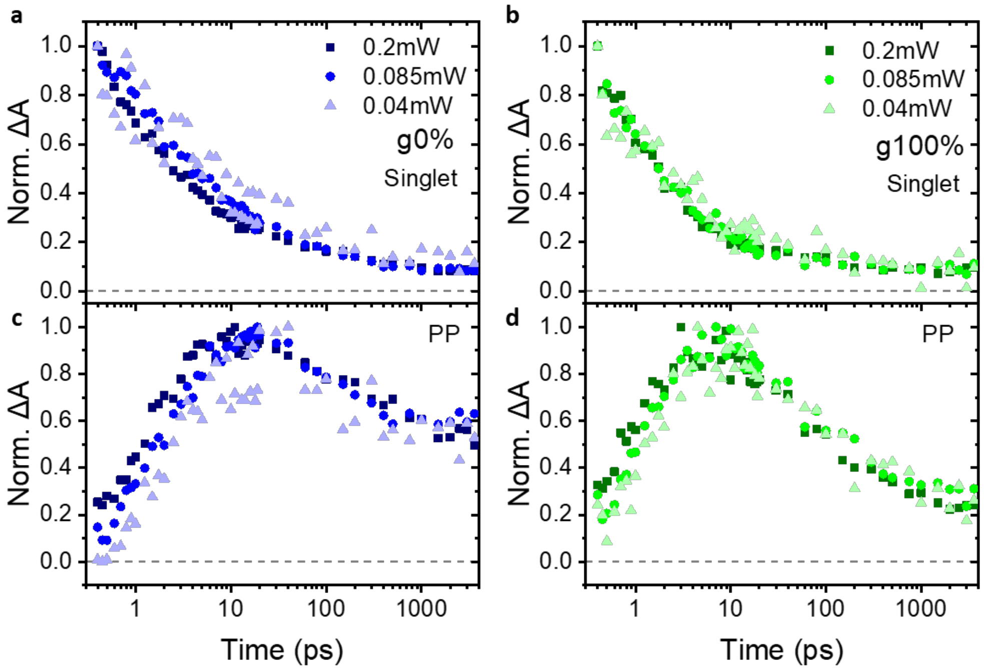

Suppl. Fig. 37: TAS fluence dependence. Kinetics of deconvoluted TAS spectra obtained via global analysis at different pump powers. **a** and **b** show the kinetics of the singlet signals for g0% and g100%, respectively. **c** and **d** show the kinetics of the polaron pair (PP) signals for g0% and g100%, respectively.

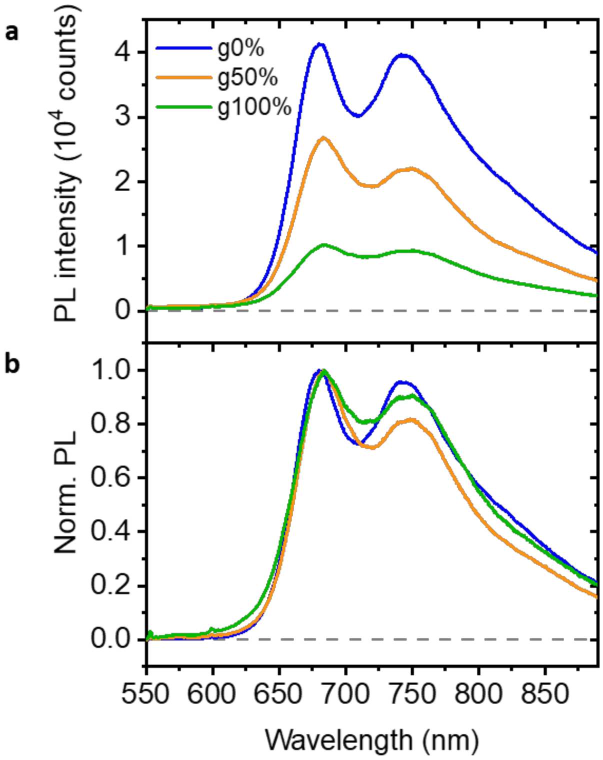

Suppl. Fig. 38: Photoluminescence spectra. a Absorption-corrected photoluminescence (PL) spectra of the g0%, g50% and g100% polymers. Samples were excited at 514 nm. **b** Normalised PL spectra.

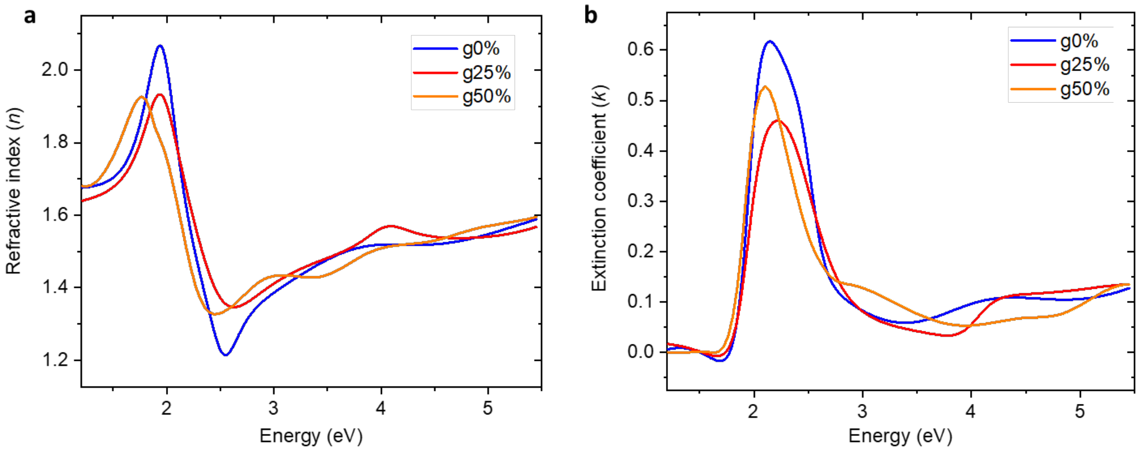

Suppl. Fig. 39: Ellipsometry. a Refractive index (*n*) and **b** extinction coefficient (*k*) of the g0%, g25% and g50% polymers measured with ellipsometry.

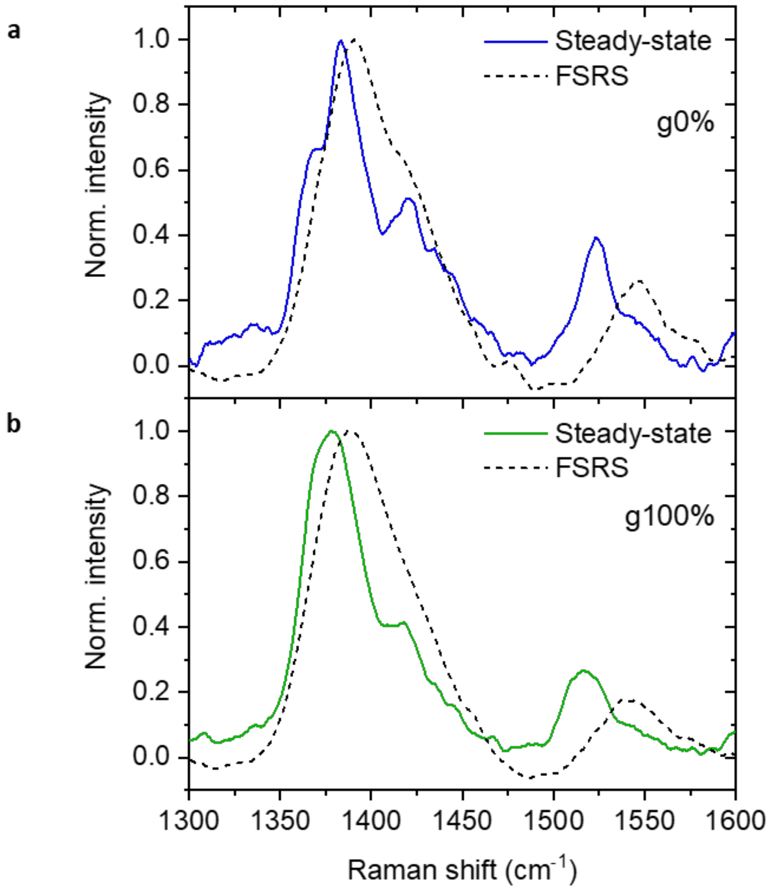

Suppl. Fig. 40: Ground-state Raman comparisons. Comparison of the thin-film ground-state Raman spectra measured in the steady-state (Figure 3) and during the FSRS measurements for the **a** g0% polymer and the **b** g100% polymer. During the FSRS measurements, a ground-state Raman spectrum is collected at each pump-probe delay when the Raman pump and white light probe overlap without the actinic pump. The ground-state Raman spectra shown here were taken at a 0 ps pump-probe delay. The ground-state Raman spectra taken during FSRS show a slight upshift in wavenumber for both the g0% and g100% polymers, which is due to the imperfect FSRS calibration.

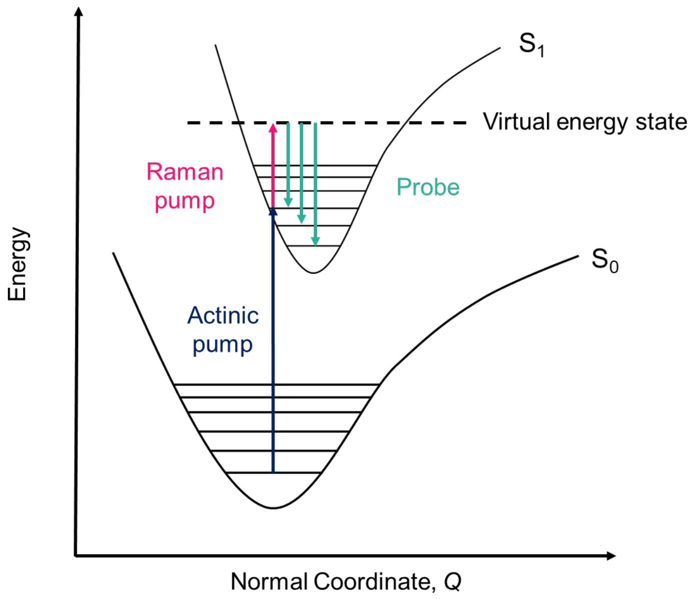

Suppl. Fig. 41: Vibrational cooling. Schematic demonstrating why shifts to higher wavenumbers are seen in ES FSRS peaks. The polymer starts in the ground state (S_0) and is excited to the S_1 state by the actinic pump. Excitation can happen to the lowest vibrational state or higher vibrational states. Stimulated Raman is then induced by the Raman pump. As the higher vibrational energy states decay to the minimum of the S_1 state, the Raman peak shifts as the energy gap to the virtual excited state widens.

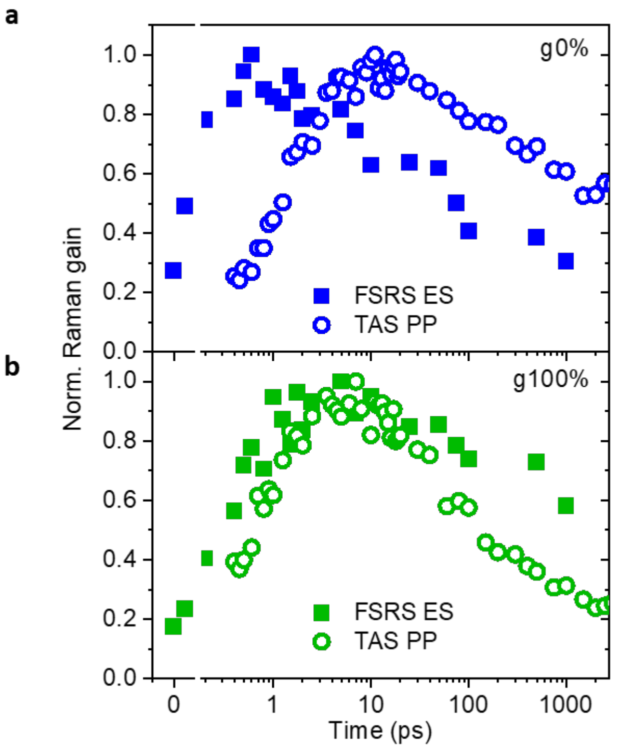

Suppl. Fig. 42: TAS and FSRS kinetics. Overlapped kinetics of formation of the TAS PP signal (open circles) and FSRS ES peak (squares) for **a** the g0% polymer (blue) and **b** the g100% polymer (green). The FSRS ES peak of the g0% polymer grows in earlier than the TAS PP signal, suggesting that structural changes in the g0% polymer are mostly associated with formation of the S_1 state. For the g100% polymer, growth of the FSRS ES peak and TAS PP signal correlate well, suggesting that PPs form during structural relaxation of the polymer.
